# Identifying Rodent Resting-State Brain Networks with Independent Component Analysis

**DOI:** 10.3389/fnins.2017.00685

**Published:** 2017-12-12

**Authors:** Dusica Bajic, Michael M. Craig, Chandler R. L. Mongerson, David Borsook, Lino Becerra

**Affiliations:** ^1^Department of Anesthesiology, Perioperative and Pain Medicine, Boston Children's Hospital, Boston, MA, United States; ^2^Center for Pain and the Brain, Boston Children's Hospital, Boston, MA, United States; ^3^Department of Anaesthesia, Harvard Medical School, Harvard University, Boston, MA, United States

**Keywords:** BOLD, fMRI, ICA, MRI, rs-fMRI, protocol, resting-state networks, review

## Abstract

Rodent models have opened the door to a better understanding of the neurobiology of brain disorders and increased our ability to evaluate novel treatments. Resting-state functional magnetic resonance imaging (rs-fMRI) allows for *in vivo* exploration of large-scale brain networks with high spatial resolution. Its application in rodents affords researchers a powerful translational tool to directly assess/explore the effects of various pharmacological, lesion, and/or disease states on known neural circuits within highly controlled settings. Integration of animal and human research at the molecular-, systems-, and behavioral-levels using diverse neuroimaging techniques empowers more robust interrogations of abnormal/ pathological processes, critical for evolving our understanding of neuroscience. We present a comprehensive protocol to evaluate resting-state brain networks using Independent Component Analysis (ICA) in rodent model. Specifically, we begin with a brief review of the physiological basis for rs-fMRI technique and overview of rs-fMRI studies in rodents to date, following which we provide a robust step-by-step approach for rs-fMRI investigation including data collection, computational preprocessing, and brain network analysis. Pipelines are interwoven with underlying theory behind each step and summarized methodological considerations, such as alternative methods available and current consensus in the literature for optimal results. The presented protocol is designed in such a way that investigators without previous knowledge in the field can implement the analysis and obtain viable results that reliably detect significant differences in functional connectivity between experimental groups. Our goal is to empower researchers to implement rs-fMRI in their respective fields by incorporating technical considerations to date into a workable methodological framework.

## Introduction

### Definition of method

Functional magnetic resonance imaging (fMRI) is one of the most commonly used neuroimaging research tools today, due to its non-invasiveness, high spatial resolution relative to other functional imaging methods, and ability to perform longitudinal studies. The technique measures intrinsic low-frequency fluctuations in the blood oxygen level dependent (BOLD) signal, as a putative index of neuronal activity (Logothetis, 2003; Raichle and Mintun, 2006). Resting-state functional magnetic resonance imaging (rs-fMRI) refers to fMRI data acquired in the absence of controlled stimuli or an explicit task. Mapping temporal covariance of BOLD signal between distinct brain regions (i.e., functional connectivity) reveals consistent patterns of large-scale functional networks termed resting-state networks (RSNs) (Buckner et al., 2013). Also known as functional connectivity magnetic resonance imaging, this technique has been utilized to investigate the effects of various drugs and neurological disorders on resting-state functional brain networks (Fox and Greicius, 2010).

### Resting-state functional connectivity in rodents

An important additional advantage of rs-fMRI is its role as a translational neuroimaging tool. Despite its exponential application in human research, comparatively few studies have applied rs-fMRI in rodent models. Mechanistic studies of rodent physiology provide mounting empirical support of rs-fMRI BOLD signal as a surrogate of underlying neuronal activity (Pan et al., 2011; Raichle, 2011; Bruyns-Haylett et al., 2013; Thompson et al., 2013, 2014). Studies mapping resting-state functional connectivity in rodents thus far have identified cortical and subcortical networks analogous to those seen in humans, that were reliably reported in both rats (Pawela et al., 2008; Hutchison et al., 2010; Becerra et al., 2011b; Jonckers et al., 2011; Liang et al., 2011; Schwarz et al., 2013; Van Der Marel et al., 2013; Sierakowiak et al., 2015) and mice (Jonckers et al., 2011; Guilfoyle et al., 2013; Nasrallah et al., 2014; Zerbi et al., 2015; Sforazzini et al., 2016). Further, rodent brain networks appear to exhibit similar frequency characteristics as those identified in human subjects (Zhao et al., 2008; Magnuson et al., 2010; Williams et al., 2010). However, recent advances in rs-fMRI data acquisition (e.g., accelerated repetition times) may yet reveal inter-species differences (Kalcher et al., 2014; Gozzi and Schwarz, 2016).

Explorations using rodent rs-fMRI thus far span a broad range of topics, including psychiatric disorders such as autism (Zhan et al., 2014), schizophrenia (Errico et al., 2015), depression (Gass et al., 2014; Ben-Shimol et al., 2015), and attention deficit hyperactivity disorder (Van Der Marel et al., 2014), as well as the impact of chronic stress (Borsook and Becerra, 2011; Henckens et al., 2015), neuropathic pain (Borsook and Becerra, 2011; Baliki et al., 2014), and analgesia (Borsook and Becerra, 2011) on functional neurocircuitry. Furthermore, rs-fMRI has been successfully applied in rodent models of neurodegenerative disease to elucidate putative genetic biomarkers (Zerbi et al., 2014), characterize disease course (Shah et al., 2013; Grandjean et al., 2014) and evaluate clinical treatment efficacies (Little et al., 2012; Wang et al., 2013, 2015a,b; Shah et al., 2015). In addition, functional connectivity mapping in the setting of pharmacological exposure (Leslie and James, 2000), including drugs of abuse (Gass et al., 2013; Lu et al., 2014) and prescription medications (Schwarz et al., 2007), sheds light on drug efficacy, optimal dosage, and possible (mal)adaptive sequelae.

### Importance of rodent models in biomedical research

There is significant interest in using rs-fMRI to identify biomarkers in neurological disease and track disease progression in both humans and animal models. In human studies, genetic vs. environmental influences are difficult to disentangle for a number of reasons, including scarcity of affected individuals and infinite potential environmental confounds. Animal models circumvent many of these issues, allowing for *in vivo* manipulation of experimental variables within highly controlled environments and further allow longitudinal studies of disease evolution or modulation by intervention. In this regard, rodents are also of particular interest due to the development of transgenic lines that model pathology of human disorders (Lythgoe et al., 2003). Further, novel methods to create “humanized” rodents (carrying functioning human genes, cells, tissues, and/or organs) may lead to substantial improvement and refinement of rodent models (Scheer et al., 2015). Given that intrinsic BOLD fluctuations in rodents and humans appear to exhibit comparable frequency ranges (Williams et al., 2010), future studies using rs-fMRI in rodent models may contribute to a greater understanding of the rs-fMRI technique (e.g., efficacy of various preprocessing measures). Certainly, translational studies of functional connectivity highlight the potential of rodent models to further explore facets of induced abnormal or pathological states at different (critical) ages at the system-level investigations. For details of species-specific rs-fMRI considerations see Box 1.

BOX 1 **Species-specific considerations.**

Comparison of rs-fMRI investigations between mice and rats are not always straightforward due to species-specific differences. Most rodent rs-fMRI studies to date have been performed in rats, despite more advanced genetic manipulation techniques in mice, owing to challenges of rs-fMRI in mice (e.g., reproducibility of brain activity). Traditionally, rats are preferred for studies in pharmacological and behavioral studies (Jacob, 1999; Lazar et al., 2005). Rats are larger than mice, facilitating procedural interventions (e.g., surgical), as well as exhibit social behaviors more in line with humans in comparison to mice (Bryda, 2013). Furthermore, more is known about rat physiology (Jonckers et al., 2015). Intimate understanding of physiology is critically important for correctly processing and analyzing rs-fMRI data as BOLD signal can be influenced by factors such as CO_2_, blood pressure, heart rate, and respiratory rates. Awake rats exhibit lower heart and respiratory rates (400 beats per min; 85 breaths per min) relative to mice (600 beats per min; 150 breaths per min) (Jonckers et al., 2015). Accordingly, mice are considered more susceptible to motion artifact at the hands of increased pulsatory and respiratory forces. Such species-specific differences will have implications for acquisition, processing and interpretation of data. Additionally, human studies suggest sex steroids and timing of reproductive cycles may influence measures of functional connectivity (Weis et al., 2011). While similar work is limited in rodents, contribution of hormones should be considered in future studies of sex differences (Peper et al., 2011). In addition to physiological confounds, functional connectivity can also be altered across different levels of anesthesia and consciousness (Liu et al., 2013). For example, the anesthetic drug propofol induced dose-dependent reductions in rat thalamocortical functional connectivity (Tu et al., 2011; Liu et al., 2013). Recent emerging trend to perform non-anesthetized rs-fMRI scans in awake animals may help to eliminate such issues in the future (Becerra et al., 2011b). Processing techniques and parameters described in the present manuscript should not be applied without careful consideration of species differences (e.g., rats vs. mice). In other words, established methodology in rats should be adapted, not adopted for studies in mice.

### Common approaches to resting-state functional connectivity analysis

Two distinct analytical techniques are typically used to assess patterns of resting-state functional connectivity, which include independent component analysis (ICA; Box 2; Stone, 2002; Beckmann et al., 2005) and seed-based correlation analysis (SCA) (Biswal et al., 1995; Hampson et al., 2002). Despite inherent differences, both analyses of rs-fMRI data produce resting-state networks that are for the most part mutually consistent (Van Dijk et al., 2010). SCA is a *model-based, hypothesis-driven* method that measures BOLD response in a predetermined region-of-interest (ROI), and then generates whole-brain correlation maps reflecting functional connectivity to designated ROI. This analytical approach is optimal when activity in a specific brain ROI is thought to be modulated by an experimental condition (e.g., drug effect vs. control). Alternatively, ICA is a *model-free, data-driven* technique that analyzes whole-brain patterns of BOLD signal fluctuation and then generates maximally independent spatiotemporal components (i.e., networks) that reflect specific neuroanatomical systems (Beckmann et al., 2005; Damoiseaux et al., 2006). Probabilistic ICA (pICA) has since evolved from the original ICA model, specifically adapted for application in fMRI datasets (Beckmann and Smith, 2004). This approach is ideal for exploratory analysis and/ or when no suitable hypothesis is available (Hyvarinen, 2013). Additionally, pICA at the individual level can be used as part of data preprocessing pipelines to identify and remove non-neuronal components stemming from physiological or motion-related artifact in the data (Beckmann and Smith, 2004; Robinson et al., 2009; Erhardt et al., 2011). Increasing availability of specialized high-field animal MRI scanners (7, 9.4, 11.7, and 15 Tesla) affords the novel opportunity to integrate systems-level analyses in labs traditionally focused on brain mechanisms at molecular and cellular levels. Brain regions with correlating patterns of activity are considered to be functionally connected (Van Dijk et al., 2010). Together, rs-fMRI and pICA provide invaluable insight into distinct patterns of large-scale brain network dynamics, complementing results from lower levels of biological complexity. The development and dissemination of these techniques to laboratories studying a wide range of clinical problems has the potential to markedly accelerate translation of basic science into clinical care.

BOX 2 **Advantages and disadvantages of independent component analysis (ICA).**

***Advantages***. ICA is a model-free, data-driven analysis that decomposes complex 4D fMRI data into simpler statistically independent components. In other words, resulting components are not dependent on a model of predicted activations (unlike univariate analysis). ICA provides a means for exploratory analysis when no hypothesis is needed or available, as no prior knowledge of brain systems is required. Most often it is employed for final network analysis to measure functional connectivity between brain regions (either resting-state or event-related). Recently, the technique has been increasingly used as part of preprocessing to attenuate physiological noise contamination, creating “cleaned” rs-fMRI datasets prior to final analysis.

***Disadvantages***. Pervasive weaknesses of the probabilistic ICA model stem from its dependence on user-defined parameters (i.e., dimensionality) and permutation ambiguity of original sources. To mitigate risk of potential type I errors (i.e., false-positive), a number of additional preprocessing steps can be employed to ensure a more conservative approach. These include: band-pass filtering and inclusion of estimated motion parameters, respiratory and cardiac signals, global BOLD signal and BOLD signals in white matter and cerebral spinal fluid as additional covariates (Cole et al., 2010; Buckner et al., 2013).

This protocol concisely and comprehensively outlines all steps important for rodent rs-fMRI data analysis using pICA. We include detailed descriptions of all the necessary preprocessing steps for removal of statistical noise and the appropriate data formatting prior to final statistical analysis. As written, the present protocol is intended for non-specialists in the field of neuroscience who are interested in adding the tool of resting-state functional connectivity analysis to their research repertoire.

## Materials and equipment

### Animals

Though presented rs-fMRI data herein are acquired from rats (Sprague Dawley, Sasco; Charles River Laboratories International, Inc., Wilmington, MA, USA), this protocol could reasonably be applied for studies in mice with minor species-specific adjustments (see Box 1). All animal studies must abide by all relevant institutional and governmental regulations. All procedures of this report were performed in accordance to the United States Public Health Service Policy on Humane Care and Use of Laboratory Animals, and the guide for the Care and Use of Laboratory Animals (NIH Publication No. 15-8013, revised 2015) prepared by the National Academy of Sciences' Institute for Laboratory Animal Research. The Institutional Animal Care and Use Committee at Boston Children's Hospital approved the experimental protocols for the use of vertebrate animals illustrated as examples in this protocol.

### Reagents

#### Anesthetics

Emerging evidence suggests neurovascular coupling under anesthesia, as well as the extent and magnitude of correlated BOLD response may be affected in a drug- and dosage-dependent manner (Austin et al., 2005; Williams et al., 2010; Pan et al., 2013, 2015). As such, for the purpose of rs-fMRI, it is essential to keep anesthesia levels uniform across all animals in the study, and comparisons between studies using different anesthetics should be approached with caution. In our previous work, we used isoflurane/O_2_ 3% (vo/vol) at 1 L/min for 3 min of anesthesia induction that was followed by 1% (vol/vol) at 1 L/min for anesthesia maintenance throughout the rs-fMRI scanning. Higher levels of isoflurane have been reported to negatively affect the BOLD signal (Wang et al., 2011; Liang et al., 2015). Detailed anesthesia protocol is described in our previous work (Bajic et al., 2016). To prepare animals for scanning in non-anesthetized states, see report by Becerra et al. (Becerra et al., 2011b).

### Equipment and equipment setup

#### Small animal MRI scanner

A vendor-supplied small animal magnetic resonance imaging (MRI) scanner (horizontal magnet; field strength 4.7T or higher) is sufficient for acquiring rs-fMRI data from rodents. Our scanning was performed with a Bruker BioSpec 70/30USR 7T MRI scanner (Bruker, Billerica, MA) at the Small Animal Imaging Laboratory at Boston Children's Hospital. The signal-to-noise ratio (SNR) is often better at higher magnetic field strengths, however this can also result in greater distortions. For the explanation of rodent positioning in the scanner and nose cone fitting please refer to Box 3. For a description of the appropriate scanning parameters refer to Box 4.

BOX 3 **Safe and efficient positioning for scanning.**

**Rodent restraining device and a nose cone**. Safe and efficient positioning of an animal in the MRI scanner (and subsequent scanning) is paramount during data acquisition and requires several steps. The restraining device should consist of a flat platform to hold the animal, an incisor hook to attach the upper incisors to the device, a head restrainer with a built-in coil to restrict head movement, an ankle bar to restrict lower body movement, and a nose cone for anesthesia delivery. The nose cone should fit over the animal nose after it has been secured on the restraining device. The animal's respiratory rate should be assessed [e.g., by using the Small Animal Monitoring and Gating System (Model 1025-2-50; Instruments, Inc., San Diego, CA)]. Paper tape should be applied over the body onto the body coil to secure respiratory rate monitor, which is placed underneath the animals (below the ventral chest).

**Radio frequency coil**. We used a Bruker inner diameter of 85 mm transmit-only volume coil in combination with a Bruker *rat* brain 4-channel phase array receive_only coil (Bruker, Billerica, MA) for adult rats and a Bruker *mouse* brain 4-channel phase array receive_only coil for 2-week old infant rats. This is because the size of the rat pups at 3rd week of life (postnatal day (PD) 14–17) was equivalent to the size of an adult mouse.

BOX 4 **MRI scanning parameters.**

Although the primary focus of this protocol involves functional images, acquiring both anatomical and functional data is recommended. Traditionally, anatomical images are acquired first in the scanning sequence protocol. For our previous experiments (Becerra et al., 2011b; Bajic et al., 2016), anatomical scans were acquired with a TurboRARE sequence without fat suppression. A FASTMAP (Fast, Automatic Shimming Technique by Mapping Along Projections) shimming technique is performed to improve the homogeneity of the B0 field. High-resolution anatomical images can be acquired with a fast spin-echo sequence as follows: RARE factor 8; repetition time (TR) 4,000 ms; echo time (TE) 35 ms; voxel size = 0.078 × 0.078 × 0.5 mm^3^; 34 slices with a 0.1 mm gap; field of view = 20 × 20^2^; in-plane resolution 256 × 256 voxels; excitation pulse = 90 degrees (2.7 ms). Subsequently, a 10-min *functional* scan should be obtained with co-centered single-shot BOLD rs-fMRI time series using an echo planar imaging (EPI) sequence with the following parameters: TR = 1,000 ms; TE = 37.323 ms; voxel size = 0.313 × 0.313 × 0.75 mm^3^; 20 slices with a 0.15 mm gap; field of view = 20 × 20 mm^2^; in-plane resolution 64 × 64 voxels; 600 volumes per animal. All parameters and procedures described are reasonably generic and will work with most small animal MRI scanners.

### Computing hardware for analysis

Using a Unix-based computer is best, as FSL (see below) is precompiled for Apple Mac (Mac OS X 10.4 or higher) and PCs (running Linux virtual machines like RedHat 9, Debian/Ubuntu, Centos) (Smith et al., 2007). The computer used for the analysis should have at least a 1 GHz CPU clock, 1 GB RAM, 5 GB swap and 20 GB of free hard drive space. Using a computer cluster (multiple computers networked together) is advantageous as it can greatly reduce overall analysis time. Analyses presented herein were performed on a reconfigured Apple Mac Pro 6-Core Intel Xeon E5 (2.70 GHz) with 64 GB RAM to run OS Ubuntu 14.04.1.

### Computing software for analysis

- **Terminal window**, often referred to as the “terminal emulator,” is a text-only *window* located within a graphical user interface (GUI) that emulates a *console*. Individual commands (as described in the protocol) can be executed within the terminal window.
- **MATLAB** (MathWorks) was used herein to develop code and perform calculations.
- Software packages like **dcm2nii** and **MRICron** for preprocessing and network visualization, respectively (http://www.mccauslandcenter.sc.edu/mricro/mricron/).

**FMRIB Software Library (FSL)**. FSL is freely available software from the Analysis Group at the University of Oxford and can be installed here: http://fsl.fmrib.ox.ac.uk/fsl/fslwiki/FslInstallation. Several FSL commands will be used in this procedure to process rs-fMRI data. Independent Component Analysis (ICA) can be implemented with software packages such as **MELODIC** (http://fsl.fmrib.ox.ac.uk/fsl/fslwiki/MELODIC) from FSL. Melodic is an acronym for: **M**ultivariate **E**xploratory **L**inear **O**ptimized **D**ecomposition into **I**ndependent **C**omponents. The program uses ICA to break down 4D (length, width, height, time) data sets into statistically independent components in spatial and temporal domains. Of note, many other ICA models exist that allow for comparable functional connectivity analysis (e.g., Group ICA Of fMRI Toolbox (GIFT) ICA package in MATLAB, http://mialab.mrn.org/software/gift/; CONN: functional connectivity toolbox (Whitfield-Gabrieli and Nieto-Castanon, 2012), https://www.nitrc.org/projects/conn).

**Important note on software versions**. The latest versions of software packages should typically be used, as they are the most up to date and optimized (e.g., bug fixes). Software versions used to process data should always be disclosed for transparency. Those used to obtain presented data in this protocol are MATLAB version R2015a, FSL v.5.0, and MELODIC v.3.14.

### Procedural outline

**Figure 1:**
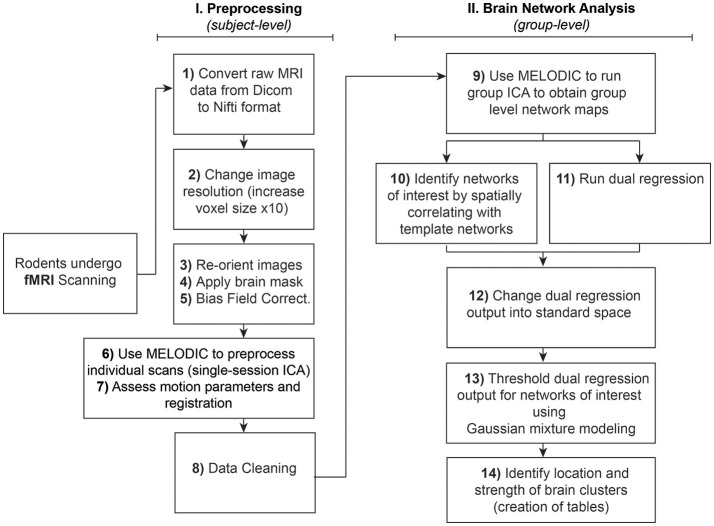
Rodent resting-state network analysis outline. Schematic outlines 14 steps for resting-state network analysis. Note that preprocessing in Step 6 should be performed at the individual level, in contrast to using group-level brain network identification in Step 9. Steps 10 and 11 can be run in any order since they are independent of each other. Note that identification of networks of interest to template networks (Step 10) assumes availability of appropriate species-specific templates. If no species-specific network templates are available for spatial correlation, one should evaluate all components qualitatively and then proceed to Step 11 (Dual Regression).

The analysis of RSNs can be divided into two major parts: **(I) Preprocessing**, which entails a series of steps performed at the subject-level aimed at preparing functional images for **(II) Brain Network Analysis** (final statistical analysis), using group pICA. Steps are summarized and outlined in Figure 1. Importantly, both preprocessing and final analyses involve a pICA run using the same Melodic interface. Instructions to set up Melodic for both subject- and group-level pICA runs are described in detail in preprocessing Step 6: Melodic Interface. However, the group-level analysis does not occur until Step 9: Network Detection via Group ICA.

## Preprocessing

Unlike task-based fMRI, studies of resting-state functional connectivity use covariance amongst time-series as the primary measure of interest, necessarily rendering the method highly sensitive to artefactual sources of signal like motion (Power et al., 2015). To address sensitivity of the technique, many spatial and temporal preprocessing steps are typically performed to minimize contamination of non-neuronal signal within the rs-fMRI data prior to final statistical analysis. Each preprocessing step is associated with unique benefits and time penalties, and can be implemented independently within the terminal window, or in combination using graphic user interfaces (GUI). Preprocessing steps for functional data (and anatomical MRI images, if available) are summarized in Figure 1 and outlined in detail below. Subsequently, preprocessed versions of rs-fMRI data are used for final statistical analysis (see section Brain Networks Analysis). Steps numbering throughout the text correspond to step numbering listed in Figure 1.

**Step 1. Convert Raw MRI Data from Dicom to Nifti Format**. Raw imaging data exported directly from the scanner are in dicom format (.dcm). In order to process MRI data using analytical tools, one must convert raw dicom files (extension _.dcm) to compressed nifti files (extension _.nii.gz; high dynamic range image file <hdrfile>). This can be achieved using the software **dcm2nii**. To convert files, one should click and drag the folder containing dicom images into the **dcm2nii** GUI.

**Step 2. Change Resolution of Functional Image**. To obtain high-resolution functional images in rodent brains, voxel sizes are much smaller than those typically seen in human brain images (e.g., 0.313 × 0.313 × 0.9 mm^3^ vs. 2 × 2 × 2 mm^3^, respectively). However, in order to process rodent MRI data using FSL tools (designed for human data), voxel sizes must be increased (e.g., by a factor of 10) so that they are comparable to human voxel dimensions. As a first step, one should identify the exact voxel size of each functional scan, and then multiply each dimension by chosen scaling factor. To alter the resolution of MR images, use the FSL command *fslchpixdim* to change the voxel size with the following format:

fslchpixdim<​hdrfile​><​xdim​><​ydim​><​zdim​>

Designated x, y, and z dimensions should be given in millimeters, while the time dimension is given in seconds. If applicable, anatomical MR images must be similarly upscaled (factor determined by voxel size).

**Step 3. Standardize Orientation of Functional Image**. Oftentimes, functional images are acquired with different orientations than the standardized anatomical image in FSLView. As a first step, one should check header information in FSLView to ensure each label correctly corresponds to the respective axis (anterior-posterior, superior-inferior, left-right). No analysis should be done using mislabeled images, as missing or incorrect header information can compromise subsequent analysis performed within FSL. To visualize functional image in FSLView and check its labels, use the *fslview* command as follows:

fslview<​hdrfile​>

After confirming axes are correctly labeled, reorient image (if necessary) into standard analyzable convention using *fslswapdim* command in the following format:

fslswapdim<​hdrfile​>−x−y−z<​hdrfile_flip​>

Listed dimensions (x, y, z) represent the new axes of functional image with respect to the old axes. Only those dimensions designated as negative values (e.g., −y and −z) are flipped in output image. Importantly, this command does not register the T1 image to any standard-space template within FSL. It simply rotates or flips the fMRI image on the three axes so that one can properly orient image to match the standard anatomical orientation in FSL. If left/right orientation was incorrectly switched, the FSL program will produce a warning message within the terminal alerting the user to the possible error. One should always visualize the fMRI image within FSLView (using *fslview* command) to confirm that rotation was successfully performed prior to moving to next step. An additional strategy to confirm and preserve the correct orientation of images after rotation is to fill a small capillary (1–2 mm in diameter) with water and attach it inferiorly to the head coil. Capillary will appear in images as a landmark for the right or left side of the brain.

**Step 4. Perform Brain Extraction**. To improve accuracy of subsequent processing steps, the functional MR image must be stripped of non-brain tissue voxels (e.g., skull). Typically, brain extraction of rodent images is carried out manually (vs. segmentation-based methods used in human studies), moving slice-by-slice through each functional image to ensure inclusion of all brain tissue and removal of obvious non-brain tissue (e.g., skull, facial structure). This can be achieved by visualizing the brain-extracted functional image overlaid on anatomical image in FSLView, using the pencil and eraser tools in the upper tool bar. Accuracy of brain extraction should be evaluated in each view—sagittal, coronal, and axial. Alternatively, an open source application called ITK-SNAP may be implemented (www.itksnap.org) (Yushkevich et al., 2006). Obtained brain extracted functional image (*hdrfile_brain*) should be used in subsequent preprocessing step.

**Step 5. Bias Field Correction**. Bias fields refer to non-uniform distributions of signal intensity across MR images. Strong bias fields in structural and/or functional MRI data can compromise registration accuracy, which relies heavily on tissue densities including gray and white matter contrast (Graham et al., 2015). This is particularly relevant to animal MRI studies that employ stronger magnetic fields resulting in stronger bias fields. Several avenues exist to correct for signal intensity inhomogeneity. FAST (FMRIB's Automated Segmentation Tool) is a fully automated method for simultaneous tissue-type segmentation and bias field estimation, available within FSL (Zhang et al., 2001). Alternative methods also exist. For example, the popular non-parametric non-uniform intensity normalization (N3) algorithm (Sled et al., 1998) and the newest version, N4ITK (Tustison et al., 2010), have been successfully employed in mouse (Lin et al., 2013) and rat (Oguz et al., 2014) MRI datasets, respectively. Bias field correction of functional data can be performed by dividing functional file by its bias field image using *fslmaths* command in FSL as follows:

$$\begin{array}{l} fslmaths<\text{​hdrfile}\_\text{brain​}>-\text{div}<\text{​estimated bias field​}>\\ \;<\text{​hdrfile}\_\text{brain}\_\text{norm​}> \end{array}$$

**Step 6. Melodic Interface**. Preprocessing of rs-fMRI data and final network detection (see section Brain Network Analysis) are performed within the Melodic interface, employing single-session (subject-level) and multi-session (group-level) pICA, respectively.

Specifically, pre-statistical preprocessing and registration in Melodic are carried out using FEAT (FMRI Expert Analysis Tool, v.6.0). Individual processing steps can be performed separately within terminal, or implemented simultaneously within Melodic GUI (with the exception of Step 8: Data Cleaning), helpfully compartmentalizing the pipeline. Instructions to setup Melodic GUI for preprocessing and network detection runs are outlined below. Following bias field correction (Step 5), the initial preprocessing Melodic run allows for quantification of motion in each scan, as well as provides an opportunity to remove artefactual processes embedded within fMRI data via single-session pICA. The latter is part of the 2-step approach referred to as ICA-based artifact removal, which capitalizes on pICA model's strength of segregating artefactual processes embedded within fMRI data into distinct components that can then be removed. To open the Melodic GUI, type “melodic_gui” (for Mac) or “Melodic” (for Linux) into terminal. Once the interface opens, setup Melodic tabs as follows:

- ***Data*** **tab**.
  - Number of Inputs: Select the total number and the actual functional images to be analyzed (“select 4D data”), as well as designate an output directory for results of the analysis. During preprocessing, the output file from Step 5: Bias Field Correction (*hdrfile_brain_norm*) serves as the input functional image. During final network analysis, “cleaned” fMRI datasets are uploaded.
  - High pass filter cutoff (s): Select the high-pass filter cutoff to define the temporal period of the scan. This protocol set the filter cutoff at 100 seconds (0.01 Hz), thereby removing BOLD signal whose temporal periods exceed the specified cutoff.
- ***Pre-stats*** **tab**. Pre-statistical processing automatically performed by Melodic GUI includes grand-mean intensity normalization of the entire 4D dataset using a single multiplicative factor. Additional modifiable options that require selection are as follows:
  - Motion correction: **Turn on** MCFLIRT (which stands for **M**otion **C**orrection by **F**MRIB's **L**inear **I**mage **R**egistration **T**ool) for preprocessing Melodic run and **turn off** for final network analysis (i.e., only perform step once). MCFLIRT corrects for changes in head position during scan acquisition in terms of rotation and translation along each axis (x, y, z). Specifically, it uses rigid body transform to realign all volumes in a given time-series to match the middle volume reference point. Realignment parameters reported by MCFLIRT can be used to assess the extent of head motion contamination present in individual time-series, as well as identify problematic motion spikes that need to be removed prior to final analysis. Spike detection can also be achieved separately within the FSL terminal, using the *fsl_motion_outliers* command.
  - Slice timing correction: Similarly, **turn on** for preprocessing Melodic run and **turn off** for final network analysis (i.e., only perform step once). Note that 2D slices in a given 3D functional volume are not acquired simultaneously during scanning (e.g., for a functional volume acquired with a TR of 4 seconds and composed of 20 slices, the last slice is obtained approximately 4 seconds after the first slice). Failure to account for differences in individual slice timing can compromise the statistical techniques used in subsequent steps, as they operate under the assumption each functional volume is acquired exactly half way through each TR. Specifically, slice timing correction uses Fourier-space time-series phase-shifting (temporal shift) to improve estimation of functional correlation between voxels in different slices (Smith et al., 2013). To correct for differences in slice timing, Melodic requires the order in which slices were obtained during fMRI data acquisition. Accordingly, the option selected here will depend on study-specific parameters. For our purposes, interleaved slice timing correction was selected from the drop down menu because volume slices were acquired in interleaved order (e.g., 0, 2, 4 … 1, 3, 5).
  - BET brain extraction: Human brain extraction tool (BET) should be **turned off** during all runs of Melodic. Rat brain extraction should be achieved manually as described in prior Step 4: Brain Extraction.
  - Spatial smoothing: Spatial smoothing is useful for enhancing the signal-to-noise ratio (SNR), which greatly improves accurate detection of true neuronal signal during final analysis. As a downside, it is known to reduce spatial resolution (Rombouts et al., 2007). Therefore, spatial smoothing should be **turned off** during preprocessing Melodic run using single-session ICA (by setting the kernel size to zero mm), and **turned on** during final network detection using group ICA (by selecting a non-zero kernel size). The degree of spatial smoothing is determined by manipulating size of the Gaussian kernel applied to fMRI data. Optimal kernel size (mm) will reduce noise without reducing valid activations. This is achieved when active brain region is larger than the size of applied smoothing kernel. Therefore, if interested in identifying small regions of activity (relative to head size), a smaller kernel works best. Alternatively, if interested in expansive patterns of brain activity (e.g., large-scale networks across the whole brain), a larger kernel size is more appropriate. Other important considerations that factor in include the rodent's brain size (e.g., pups vs. adults) and quality of fMRI data (e.g., SNR). Based on our previous work (Bajic et al., 2016), we recommend a Gaussian kernel FWHM of 0.7 mm to identify larger patterns of functional connectivity.
  - Temporal filtering: Studies to date suggest humans and rodents exhibit similar ranges of resting-state temporal frequencies (Zhao et al., 2008; Magnuson et al., 2010; Williams et al., 2010). Accordingly, current consensus holds that temporal filters applied to rs-fMRI data should be similar across species (Gozzi and Schwarz, 2016). In light of recent advances in scan sequences, our current understanding of cross-species differences may change (Kalcher et al., 2014). In practical terms, **turn on** “Highpass” (by selecting box) during preprocessing Melodic run, and **turn off** (by deselecting box) for final network detection using group ICA (i.e., only perform step once). High-pass temporal filtering will remove lower frequencies (e.g., <0.01 Hz) from rs-fMRI data whose temporal periods exceed the filter cutoff defined in *Data* tab. This will eliminate linear trends in the data, including slow temporal drifts characteristic of scanner artifacts (Feinberg et al., 2010). Alternatively, band-pass filtering (i.e., simultaneous use of high- and low-pass filters) can be used to effectively define a range of BOLD signal frequencies to be retained within rs-fMRI data, and remove frequencies that fall outside desired range. **Note** that low-pass filtering removes higher frequencies from rs-fMRI data, with oscillatory speeds above a designated threshold. Band-pass filtering must be performed separately in the terminal window using the *fslmaths* command with the –bptf option, as Melodic does not offer this. Traditionally, resting-state networks have been considered low-frequency fluctuations in BOLD signal between 0.01 and 0.1 Hz (100 and 10 seconds). Accordingly, this protocol applies band-pass filtering to retain only this range of frequencies (similar to past rodent studies (Becerra et al., 2011b; Liang et al., 2011). However, in light of emerging evidence that suggests valuable neuronal signal may be present > 0.3 Hz (Feinberg et al., 2010; Boubela et al., 2013), it may be advisable to apply highpass temporal filtering only.
- ***Registration*** **tab**. During single-session preprocessing, each rodent's functional image can be co-registered to its corresponding anatomical image in ***native space*** (i.e., coordinate system unique to individual) and/or projected into a ***standard space*** (i.e., coordinate system common to all subjects). Accordingly, select the “Main structural image” option (if applicable) to align each rodent's functional and anatomical images in native space, and/or “Standard-space” option to normalize to a standard space (e.g., template or atlas). The latter is absolutely required for group-level analysis (e.g., Step 9: Network Detection via Group ICA). Next, define the desired resampling resolution (e.g., 4 mm) and degrees of freedom (e.g., 12 dof). Robust linear (affine) registration is carried out using FLIRT (**F**MRIB's **L**inear **I**mage **R**egistration **T**ool) (Jenkinson and Smith, 2001; Jenkinson et al., 2002). Registration techniques like FLIRT rely heavily on tissue densities (e.g., gray-white-matter contrast) to accurately align images (Graham et al., 2015). Poor tissue contrast and/or spatial resolution of functional images, as well as individual variations in rodent brain size and (to a lesser degree Pan et al., 2015) morphology can result in suboptimal registration. Accordingly, it may be advisable to co-register each rodent's fMRI image to its corresponding high-resolution anatomical image in native space as an intermediary step, prior to normalization to standard space. To do so, select “Main structural image.” For further details regarding linear and non-linear transforms for brain registration, please see published work by Klein et al. (2009). In this protocol, rodent fMRI data was normalized to a study-specific rodent template based on the rodent Atlas (Paxinos and Watson, 1998), generated in-house (see **Figures 7** and **8**). Such study-specific templates can be generated from a single anatomical scan (e.g., study by Becerra et al., 2011b), reflecting the “typical” image for the study, or from many scans, reflecting the mean of each experimental group or all subjects combined. For access to the in-house template used in current work, contact Dr. Lino Becerra (lino.becerra@childrens.harvard.edu). Alternatively, pre-existing rodent MRI templates are available for researchers, including both mouse (Ma et al., 2008; Bai et al., 2012; Papp et al., 2014) and rat (Schweinhardt et al., 2003; Schwarz et al., 2006; Lu et al., 2010; Valdes-Hernandez et al., 2011; Nie et al., 2013; Wisner et al., 2016). Currently, publicly available rodent brain MRI templates in atlas space are somewhat lacking in number, with considerable variability in methodology (e.g., number of animals, data acquisition parameters and processing pipelines). Additionally, while the majority of brain templates to date are Paxinos-Watson atlas based, other rodent atlases are beginning to emerge for rats (Papp et al., 2014) and mouse brains (Mackenzie-Graham et al., 2004, 2007; Ullmann et al., 2013). Consequently, it is imperative that users understand the origins and inherent assumptions underlying preparation of chosen template/atlas if adopted from pre-existing database.
- ***Stats*** **tab**. Standard pre-ICA processing automatically performed by Melodic includes masking of non-brain voxels and voxel-wise de-meaning of the rs-fMRI data. Additional modifiable options that require selection include:
  - Variance-normalize timecourses: Select “Variance-normalize timecourses” during all runs of Melodic (default setting). Each time-series will be rescaled such that analysis is primarily influenced by voxel-wise temporal dynamics instead of a given voxel's amplitude signal. In other words, ICA places greater importance on *temporal changes* in signal within a given area, rather than the *average signal* in that area.
  - Automatic dimensionality estimation: This parameter allows one to control the ICA decomposition process, transforming fMRI data into independent components. During preprocessing, the purpose of single-session ICA is to segregate data into components so that embedded artefactual processes can be removed. Accordingly, one should select “Automatic dimensionality estimation” during preprocessing Melodic run. This instructs Melodic to objectively estimate the dimensionality for each fMRI file based on the quantity and quality of data therein, facilitating ICA convergence stability. During final brain network analysis, one may choose to enforce a uniform dimensionality across all runs of group ICA by deselecting “Automatic dimensionality estimation” and designating the desired number of output components. Currently, there is no consensus on how to determine optimal dimensionality. Higher dimensionalities increase incidence of component “splitting” into sub-components, which has been argued to provide more biological detail and speculatively reflect functional hierarchy (e.g., brain networks split into sub-networks) (Fransson et al., 2007; Smith et al., 2009, 2013). While this tends to increase functional homogeneity within each component (desirable), higher dimensionalities also tend to generate noisier associated timecourses (undesirable) as fewer and fewer time-series are averaged together (Smith et al., 2013). Further, too high a dimensionality may compromise comparative analyses due to topological variability between individual animal scans (Smith et al., 2013). Ultimately, optimal dimensionality will depend on study-specific quality and quantity of rs-fMRI datasets, as well as the intent of analysis (Smith et al., 2013). Aim of group analysis in present protocol was to achieve a reasonable balance between component combination and splitting, decomposing rs-fMRI data into interpretable components and sub-components. Accordingly, ICA herein was set to extract 40 independent components (similar to previous investigations; Hutchison et al., 2010; Liang et al., 2011), which resulted in an appropriate decomposition.
  - Single-session ICA: During preprocessing, select “Single-session ICA” from the drop down menu to analyze individual fMRI data files separately. This maintains session/subject-specific variation that will improve detection of artifacts, which can have high inter- and intra-subject variability. To perform group ICA, one should select “Multi-session temporal concatenation.” This instructs Melodic to concatenate (i.e., link together) individual subject's time-series to form a single multi-subject time-series that can then be analyzed by ICA, resulting in group-level component spatial maps that reflect large-scale patterns of functional connectivity in the sample. Group ICA effectively defines functional networks of interest, particularly useful for group-wise comparisons. Importantly, this approach does not assume temporal response patterns are uniform across the sample, allowing associated timecourses to differ while constraining spatial maps.
- ***Post-stats*** **tab**: Leave all options at their default setting for all runs of Melodic. Specifically, the “Threshold IC maps” option will be automatically set at 0.5, meaning extracted component spatial maps are thresholded with the alternative hypothesis tested at *P* > 0.5 for activation (signal) vs. null (noise).

Once setup is finished, press the “Go” button in the bottom left corner of the Melodic GUI to run the analysis. Time requirements for both single- and multi-session analyses are highly dependent on the number of animals included in the analysis.

**Figure 2:**
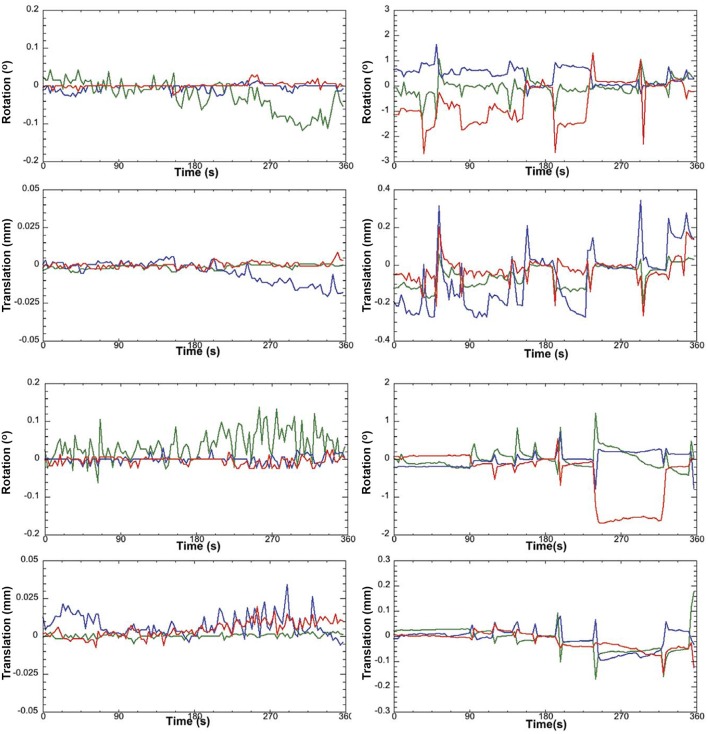
Awake rat motion assessment. The motion parameters for 2 typical rats (Left column) in the study and the 2 rejected rats (Right column) are displayed. Green Line, translation/rotation X-axis, Blue Y-axis, and Red Z-axis. Figure reprinted with permission from Becerra et al. (2011b) study in adult rats.

**Figure 3:**
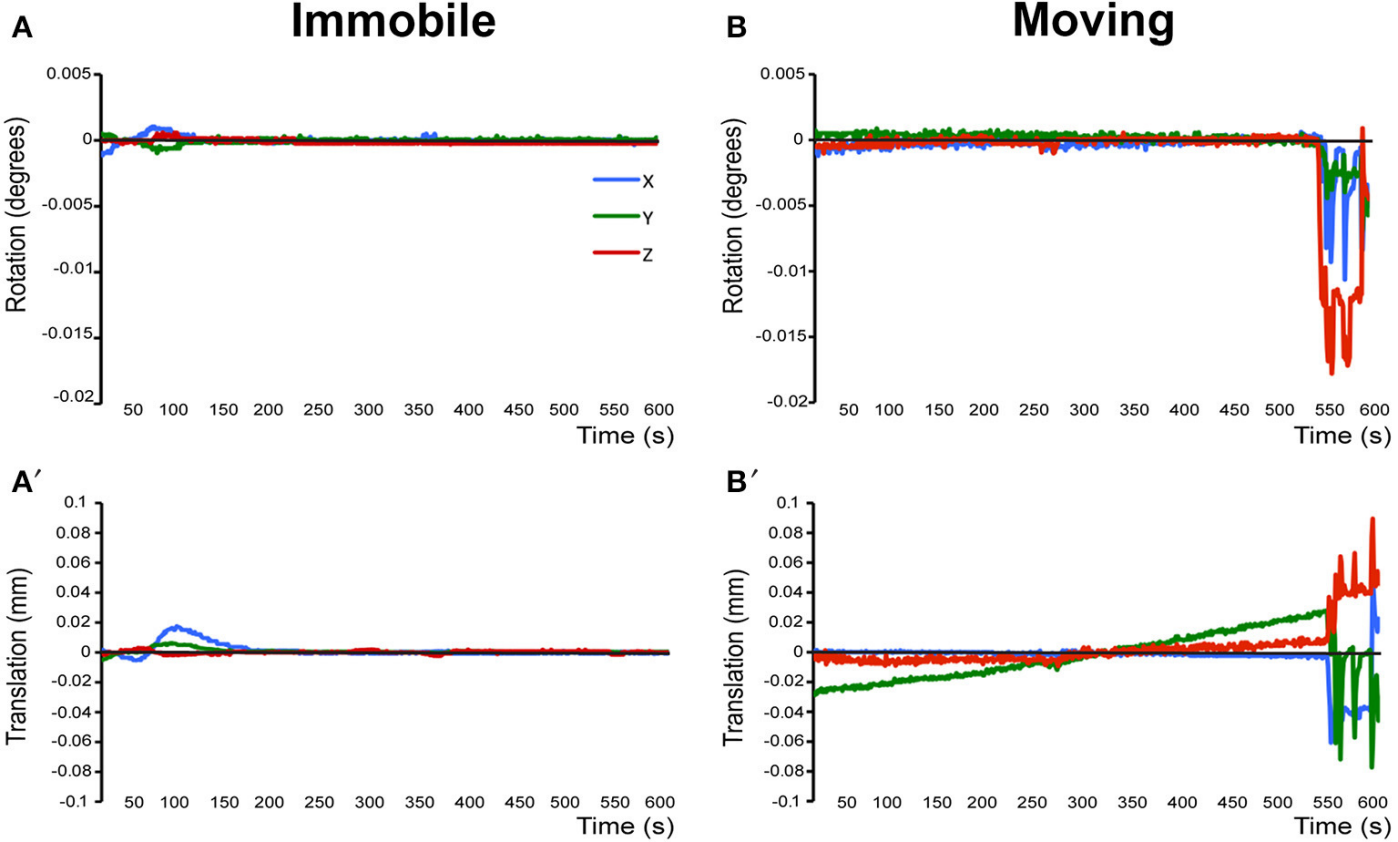
Assessment of motion in lightly anesthetized infant rats during imaging. **(A,A**′**)** display the rotation (in degrees) and translation (in mm) for an immobile 2-week-old rat during MRI, respectively. Rotation is a rigid body movement and refers to the movement of the head around a center point. Translation is every point on the head moving a constant distance in a specific direction. The immobile rat's head did not rotate more than 0.005 degrees or moved more than 0.02 mm. This is an acceptable amount of movement for the group ICA. **(B,B**′**)** illustrate rotation and translation of an infant rat that moved during the scanning, which lead to a motion-related imaging artifact. As a result, data obtained from this animal was excluded from the group ICA. Blue X line, horizontal axis; Green Y line, vertical axis; Red Z line, longitudinal axis of the scanner. Figure reprinted with permission from past study (Bajic et al., 2016) in infant rats.

**Figure 4:**
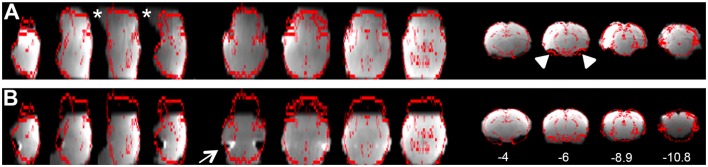
Examples of registration. **(A)** Illustrates representative individual animal functional-to-standard registration of the rat. The gray image is individual resting-state fMRI data while the red contour represents the outline of an adult anatomical atlas as reported by FSL output. First 4 columns are in the axial view; the middle 4 are in the sagittal view; last 4 columns are in coronal view. A common expected artifact is seen in the ventral regions of the rat brain [near ear canals (Schwarz et al., 2006)] and is noted in the second coronal section (*arrowheads*). Distortions noted in the ventral parts of the brainstem were noted in the caudal region of the brainstem (*stars*). **(B)** Shows an extreme example of the erroneous registration when the individual resting-state fMRI data image is rotated 180 degrees to the anatomical template. There is an obvious mismatch of registration that clearly implicates flipped data registration as seen in the temporal regions (first axial section; *arrow*). Obviously, such case of erroneous registration should not be included in subsequent analysis. Numbers below coronal slices represent distance from Bregma (mm). Left hemisphere of the brain corresponds to the right side of the image. Section with Bregma of 0 mm corresponds to Panel 17 of Rat Brain Atlas (Paxinos and Watson, 1998).

**Step 7. Review Melodic Report (Critical Step)**. Melodic generates a folder of results for each file run through analysis, including a convenient Melodic report (report.html) that contains a summary of results. After single-session pICA analysis is finished for a given functional file, one should open its Melodic report and evaluate (1) MCFLIRT motion parameters and (2) registration of functional image to standard space as follows:

- **Pre-stats tab: MCFLIRT motion parameters**. Individual time-series should always be assessed for motion contamination prior to deciding whether fMRI data should be included or excluded in final group analyses. Motion assessment is particularly important in cases of imaging awake subjects (Figure 2). However, absence of motion must be confirmed even when imaging was performed on anesthetized animals (Figure 3; see also Figure 3 Bajic et al., 2016), as even miniscule changes in head position can introduce false statistical significance (Power et al., 2012). To evaluate motion, inspect MCFLIRT graphical results of realignment parameters for any abrupt changes in head position (i.e., motion spikes) throughout the animal's time-series. Each rigid body transform performed by MCFLIRT is defined by six parameters: estimated rotational (in degrees) and translational (in mm) displacement along the three axes (x, y, z). These six parameters are condensed by MCFLIRT into a single vector referred to as the root-mean-squared (RMS) displacement, expressed in mm (rotational displacements are converted to mm). This is a summary statistic, describing total head position change in terms of *absolute* and *relative* measures. Specifically, absolute RMS displacement describes head position for a given volume with respect to a reference time point (e.g. middle volume in the time-series), useful for identifying gradual shifts in head position. Relative RMS displacement, often referred to as frame-wise displacement (FD), describes head position for a given volume relative to the subsequent volume in BOLD time-series (Power et al., 2012). Alternatively, DVARS (derivative of RMS variance over voxels) can be used as a highly sensitive index of motion, describing changes in signal intensity across the entire brain image relative to the subsequent volume in time-series (Smyser et al., 2013; Gao et al., 2014, 2015a,b; Power et al., 2014). DVARS has also been strongly correlated with relative RMS displacement (Power et al., 2012; Satterthwaite et al., 2013). An appropriate definition of excessive motion (unsalvageable volume) will depend on the scanning parameters used to acquire rs-fMRI data (e.g., length of TR), and cannot simply be adopted from preexisting literature (Power et al., 2014, 2015). In this protocol, excessive motion was defined as estimated rotation > 0.005 degrees and/ or translation > 0.02 mm along any axis, as well as RMS displacement exceeding more than half a voxel size. If definition of excessive motion is met, motion censoring (i.e., data scrubbing via targeted volume removal) may be used to salvage time-series (see sections Troubleshooting and Motion Censoring).
- **Registration tab**. Ensure proper alignment of functional images to the structural template. Common expected minimal artifacts are well described in the literature (Schwarz et al., 2006) and occur in the ventral regions of the brain near ear canals (Figure 4A). See also Figure 2 in Bajic et al. (2016). An example of an extreme case of erroneous registration is illustrated in Figure 4B when the image is flipped 180 degrees in relation to template. Precise registration is crucial for fMRI analysis, as well as any other image analyses that require functional-to-structural alignment (e.g., structural and diffusion image analysis). If individual registrations are inaccurate, further statistics at a structural or group level will likely be inaccurate. Poor registration may be salvageable (see sections Troubleshooting and Registration).
- **ICA tab: Component Classification**. As previously mentioned, ICA-based artifact removal involves an initial preprocessing run of pICA to decompose 4D fMRI data into independent components. During pICA, the dimensionality (i.e., number of components) of each animal's fMRI data was objectively estimated. As a result, estimated dimensionalities listed in the Melodic report will likely vary between fMRI files. An inherent advantage of the ICA model is that it helpfully segregates signal embedded within fMRI data based spatiotemporal characteristics, such that signals arising from a similar source are more likely to group together in a given component (e.g., motion-related, blood vessels, venous sinuses, neuronal). Components identified as noise can later be removed from fMRI data (see Step 8: Data Cleaning). However, the ICA model does not classify signal origins isolated in each extracted component (e.g., signal or noise). Consequently, components must be classified following analysis as either “good” (i.e., predominantly signal) or “bad” (i.e., predominantly noise) by evaluating all spatiotemporal features of each component in a hierarchical manner. Component classification can be achieved manually (by human rater) or by automated or semi-automated classifiers (e.g., FIX (FMRIB's ICA-based X-noiseifier); see Step 8b. Automated Data Cleaning). Neither method is perfect. While currently considered the golden standard (McKeown et al., 1998; Moritz et al., 2003; Kelly et al., 2010), manual classification is time-consuming (increasing risk of fatigue and error), and critically dependent on operator expertise and inter-rater reliability. Further, while classifiers are often described as fully automated, their performance should realistically be monitored for accuracy and consistency within and between experimental groups. Accordingly, a solid understanding of typical spatiotemporal features associated with neuronal and artefactual components is essential, regardless of the method. Refer to recent work by Zerbi et al. (2015) for extensive illustrations of group-level signal and noise components identified in rodents (Zerbi et al., 2015). Additionally, the recently published work by Griffanti et al. (2017) provides a comprehensive “how-to” guide on component classification in human subjects that can reasonably be adapted to other species (Griffanti et al., 2017). If performing classifications by purely manual methods, raters must evaluate each component from a given ICA run and compile a list of recorded classifications (neuronal, artifact, unknown component).

**Important Points of Preprocessing**. Motion spikes within the fMRI time-series and/ or poor registration will compromise the accuracy of subsequent analyses. If excessive motion and/ or unsatisfactory registration cannot be rectified at the single-session level (see section Troubleshooting), it is advisable to exclude the afflicted animal dataset from group-level processing and analysis.

**Step 8. Data Cleaning**. The second stage of ICA-based artifact removal, referred to as “data cleaning,” allows for the removal of unique variance associated with artefactual sources of signal (e.g., motion and physiological). Specifically, components identified as predominantly noise during review of the Melodic report (Step 7c: ICA tab) are removed, effectively creating “cleaned” 4D fMRI datasets (Smith et al., 2011, 2013). Data cleaning can be achieved *manually* (by human rater) or by *automated* classifiers.

- **Manual data cleaning**. For smaller sample sizes or unique patient populations, it is usually advisable to classify all components by hand (Griffanti et al., 2017). Manual de-noising of fMRI data can be performed within terminal using the command *fsl_regfilt* in the following format:
  $$\begin{array}{l} fsl\_regfilt-\text{i }<\text{​filtered}\_\text{func}\_\text{data​}>-\text{o}<\text{​​denoised}\_\text{data​​}>\\ \;-\text{d}<\text{​filtered}\_\text{func}\_\text{data}.\text{ica/melodic}\_\text{mix}>\\ \;-\text{f "}1,2,3\ldots " \end{array}$$
  Option –i designates the input file containing preprocessed fMRI data (*filtered_func_data*), located within the rodent's preprocessing single-session Melodic output directory. Option –o designates the output file containing denoised fMRI data (*denoised_data*). Option –d stands for “design,” and designates subsequent file listed as containing the corresponding Melodic mixing matrix (*melodic_mix*). Because the design matrix file is in directory below the rodent's single-session melodic output directory, it is necessary to define the pathway in command followed by a slash (*filtered_func_data.ica/melodic_mix*). Option -f designates list of unwanted components (“1,2,3…”) to be filtered out of the regression model. For example, if ICA components 3, 10, 15 and 55 are manually classified as “bad” when reviewing the Melodic report (see Step 7c: ICA tab), the –f option would be formatted as –f “3,10,15,55”. Importantly, double quotes must encompass the list in order for the entire list of components is passed to Melodic. Note, while the Melodic report.html begins component numbering at one, FSL software and actual Melodic output files begin numbering at zero (e.g., component 4 in Melodic report is defined as component 3 by FSL).
- **Automated data cleaning**. Researchers using large sample sizes as in rodent studies may benefit from the advanced automated approaches that are now emerging. Notably, the recently developed fusion classifier^1^ FIX (FMRIB's ICA-based X-noiseifier) employs ensemble learning, evaluating over 180 spatiotemporal features to arrive at a final weighted classification. Its application in numerous human rs-fMRI studies yield promising results, particularly when trained on study-specific datasets (>95% accuracy, with >99% using trained classifier) (Smith et al., 2013; Griffanti et al., 2014, 2015; Salimi-Khorshidi et al., 2014; Feis et al., 2015). To our knowledge, only one study conducted by Zerbi et al. has implemented FIX to evaluate rodent rs-fMRI datasets, demonstrating high levels of accuracy comparable to results obtained in human studies (Zerbi et al., 2015). The study-specific mouse training datasets and trained FIX classifier describe by Zerbi et al. (2015) are now publicly available (Central.xnat.org; Project ID: CSD_MRI_MOUSE; mice ID's: 1366, 1367, 1368, 1369, 1371, 1378, 1380, 1402, 1403, 1404, 1405, 1406, 1407, 1411, and 1412).

## Brain network analysis

**Step 9. Use Melodic to Perform Network Detection via Group ICA**. Preprocessed and cleaned fMRI data will now be run through group ICA, as part of final statistical analysis. Refer to Step 6 for instructions on how to setup the Melodic interface (and tabs therein) to perform group-level analysis of data. Group-level components obtained from this step reflect large-scale patterns of functional connectivity in a given sample.

**Figure 5:**
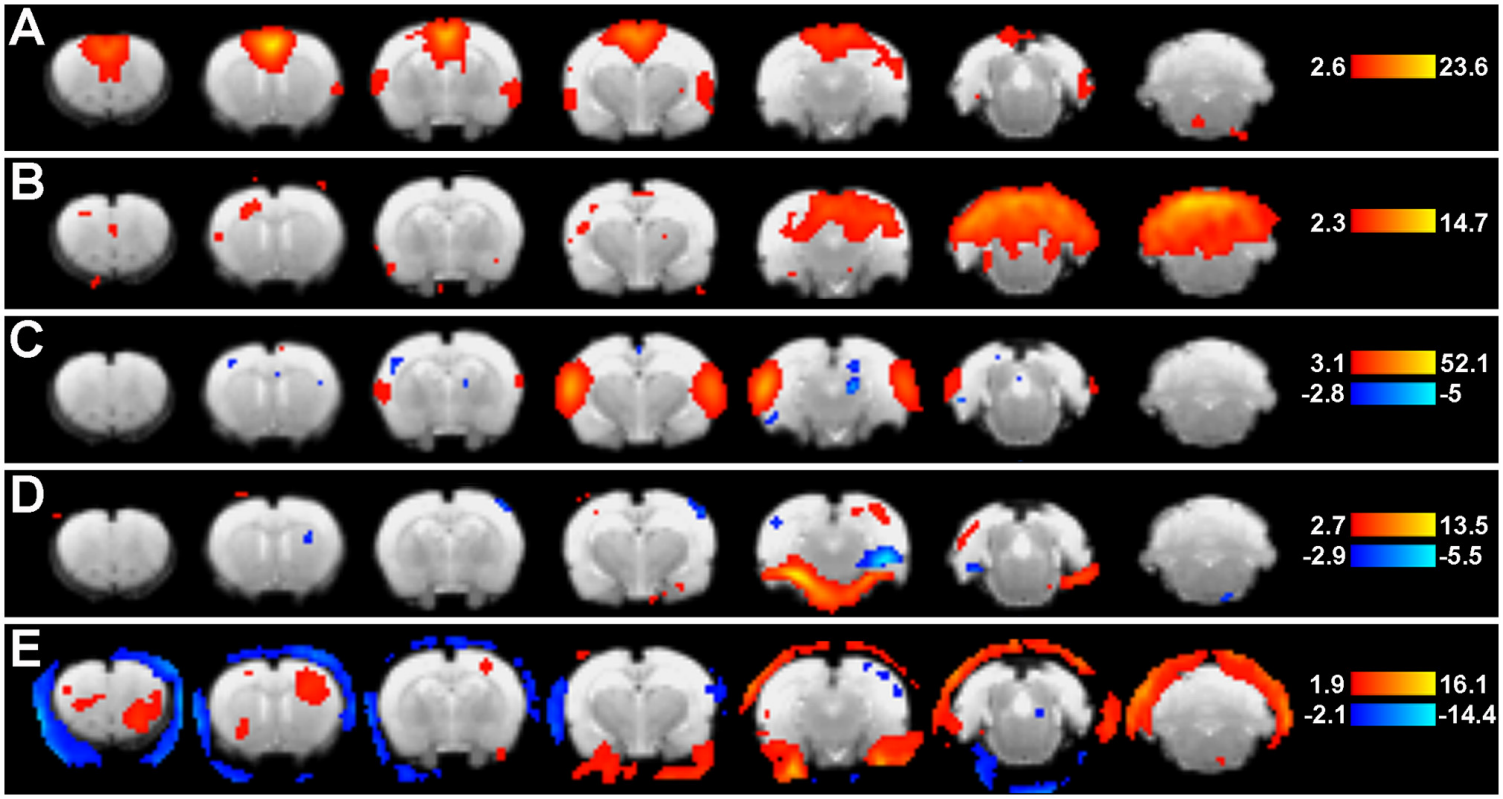
Group ICA spatial maps. Figure shows representative group-level component spatial maps extracted from group ICA (Step 9: Network Detection via Group ICA) as they appear in the Melodic report, including pre-determined statistical thresholds (warm colors reflect positive z-scores, while cold colors reflect negative). Putative neural networks **(A–C)** show coherent BOLD signal predominantly arising from gray matter, while non-neuronal artefactual components **(D,E)** show a large degree of edges. Left side of image corresponds to right side of brain.

**Figure 6:**
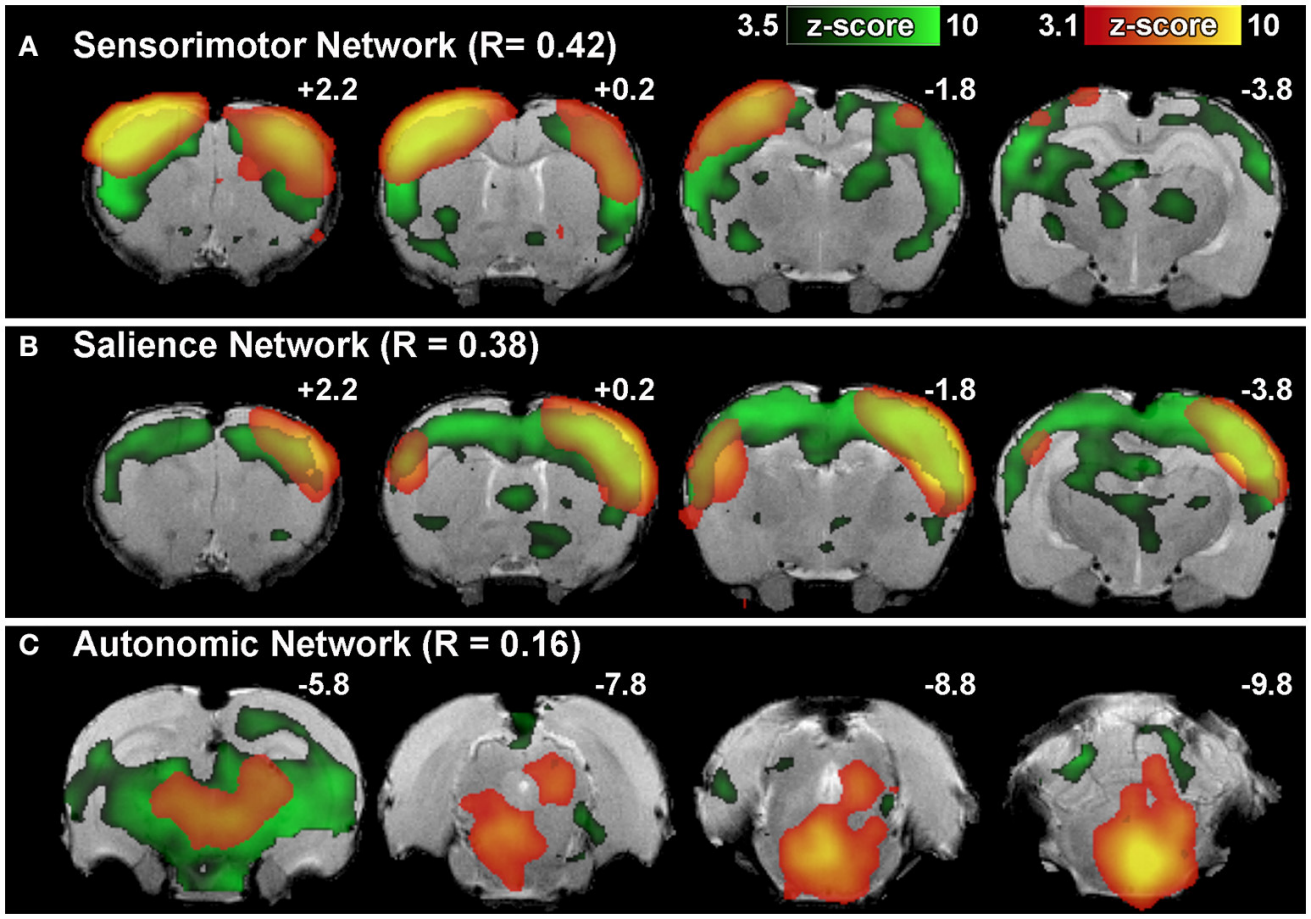
Brain network identification via spatial correlation. Figure illustrates representative group-level component spatial maps extracted from group ICA for **(A)** Sensorimotor, **(B)** Salience, **(C)** Autonomic Networks (red-yellow) overlaid on spatial maps of correlated template networks (green). Spatial correlation R > 0.20 between individual components and template network(s) is sufficient to identify potential functional networks of interest amongst the full set of extracted group-level components. Components that do not meet criteria for network classification based on set of template networks (e.g., R-values <0.20 for all templates) may still contain biologically relevant brain activity (as in the example of Autonomic Network). Numbers above each coronal section represent distance from Bregma (in mm). Left side of image corresponds to right side of brain.

**Figure 7:**
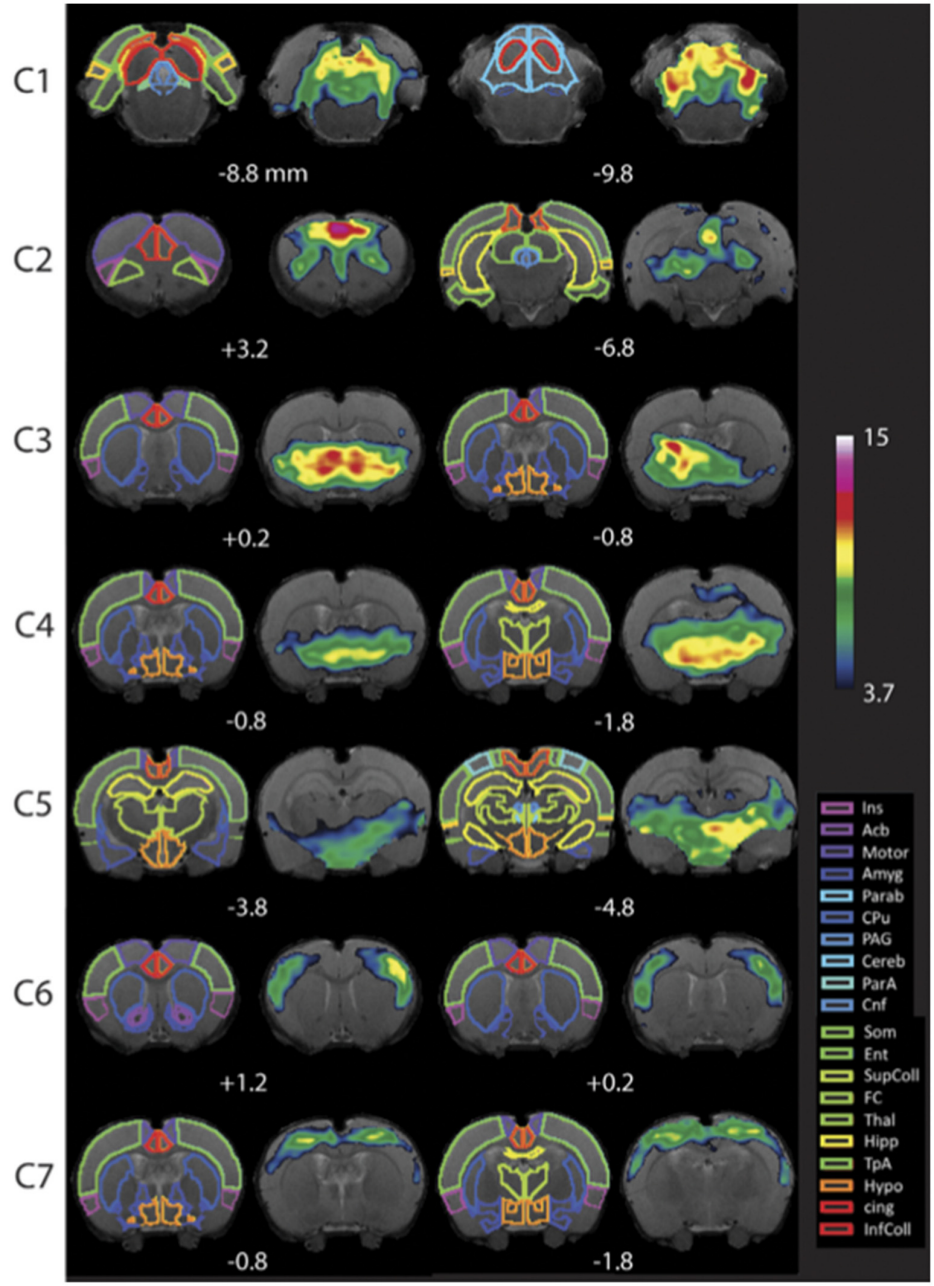
Resting-state networks in awake rats. Complete maps for Components (C1–C7). All components have been thresholded according to a mixture model approach. See Methods from Becerra et al. (2011b) for details. The atlas is based on the Paxinos and Watson Atlas (1998). Abbreviations: Ins, Insula; AcB, Nucleus Accumbens; Motor, Motor Cortex; Amyg, Amygdala; Parab, Parabrachial; CPu, Caudate-Putamen; PAG, Periaqueductal Gray; Cereb, Cerebellum; ParA, Parietal Association Cortex; Cnf, Cuneiform Nucleus; Som, Somatosensory Cortex; Ent, Entorhinal Cortex; SupColl, Superior Colliculus; FC, Frontal Cortex; Thal, Thalamus; TpA, Temporal Association Cortex; Hypo, Hypothalamus; cing, Cingulate Cortex (anterior and retrosplenial); InfColl, Inferior Colliculus. Figure reprinted with permission from Becerra et al. (2011b).

**Figure 8:**
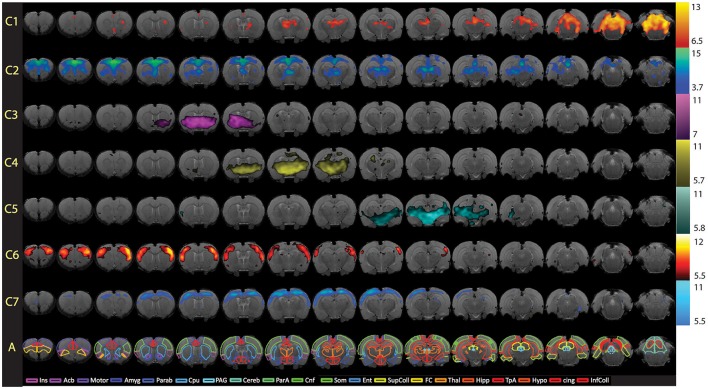
Full spatial maps of resting-state networks in awake rats. Components (C1–C7) are ordered according to their reproducibility degree. Component 1 has significant cerebellar structures; Component 2 includes medial and lateral cortical structures resembling the human default mode network; Component 3 includes a basal-ganglia-hypothalamus network; Component 4 encompasses basal-ganglia-thalamus-hippocampus circuitry; Component 5 represents an autonomic pathway; Component 6 represents the sensory network; and Component 7 groups interoceptive structures to form a network. All components have been thresholded according to a mixture model approach. See Methods section for details. The atlas is based on the Paxinos and Watson Atlas (1998). *A*bbreviations: Ins, Insula; AcB, Nucleus Accumbens; Motor, Motor Cortex; Amyg, Amygdala; Parab, Parabrachial; CPu, Caudate-Putamen; PAG, Periaqueductal Gray; Cereb, Cerebellum; ParA, Parietal Association Cortex; Cnf, Cuneiform Nucleus; Som, Somatosensory Cortex; Ent, Entorhinal Cortex; Sup Coll, Superior Colliculus; FC, Frontal Cortex; Thal, Thalamus; TpA, Temporal Association Cortex; Hypo, Hypothalamus; cing, Cingulate Cortex (anterior and retrosplenial); Inf Coll, Inferior Colliculus. Figure reprinted with permission from Becerra et al. (2011b).

**Step 10. Evaluate Group-Level Components To Identify Networks Of Interest**. Following group ICA, group-level components should be carefully inspected (Figure 5) in order to distinguish noise components from networks of interest (i.e., those reflecting biologically relevant neuronal signal). Group ICA output file containing extracted components (*melodic_IC*) is stored in the Melodic output directory. Components can be evaluated by qualitative and/ or quantitative methods to identity representative brain networks. *Qualitative* measures entail visual inspection of temporal (timecourse), spectral (powerspectrum) and spatial characteristics (spatial maps) to identify components of interest. In order to view each component overlaid on standard-space template within FSL (using *fslview* command), the dimensions of functional files must be converted to match that of the anatomical template. This can be achieved using *flirt* command. Once dimensions are equivalent, components can be viewed superimposed on anatomical template image, which is helpful for localization of BOLD signal. Alternatively, *quantitative* methods can involve measuring spatial correlation (Pearson's R) between extracted components and templates of canonical large-scale rodent networks (e.g., default-mode vs. visual network). While there are currently no standardized rodent templates, one can use network templates made available by previously published studies (e.g., mouse Zerbi et al., 2015, or rat templates). Our group uses 7 template networks reliably identified in adult rat brain, reported by Becerra et al. (2011b). Highest correlation (i.e., degree of spatial overlap) between component and a given template is helpful in discerning the probable identify of component. While the metric is not without controversy, spatial correlation can be helpful in directing attention to definite components of interest. Correlated spatial overlap *R* > 0.20 with a template network is sufficient to identify potential candidate networks of individual components (Figures 6A,B). Higher statistical thresholds (e.g., *R* > 0.40) would identify component identities with greater certainty, however, this would necessarily risk exclusion of biologically relevant brain activity such as network sub-components, as well as relevant components excluded due to potential topological variability across subjects, groups and/or strains (e.g., Figure 6C). Accordingly, studies should not rely on this methodology in a confirmatory capacity or as the sole means of identifying components of interest, as this engenders risk of missing valuable components. Meaningful evaluation and interpretation of group ICA spatial maps will require thorough knowledge of previously reported rodent RSN topologies to inform component classifications (see also Figures 7 and 8). Regardless of chosen measure, only ICA components identified as brain networks of interest should be processed later in Steps 12 and 13 related to Gaussian mixture modeling.

**Step 11. Analyze All Group Level Components Using Dual Regression**. As previously mentioned, component spatial maps extracted from group ICA reflect group-level patterns of functional connectivity in the sample. Dual regression can now be performed to identify associated timecourses (stage 1) and spatial maps (stage 2) within individual subject's rs-fMRI data that correspond to group-level components (Filippini et al., 2009). Specifically, dual regression probes intra-group consistency of functional connectivity patterns in order to provide measures of intra-group differences. This approach, referred to as *multiple linear regression*, is suggested to be more reliable than alternative back-projection methods, which can produce false statistical significance (Filippini et al., 2009). Further information regarding the technical aspects of dual regression can be found on the FSL website (http://fsl.fmrib.ox.ac.uk/fsl/fslwiki/DualRegression). For detailed descriptions of common multi-subject experimental designs and instructions on how best to setup group contrasts (viz. group comparisons), refer to the FSL website (https://fsl.fmrib.ox.ac.uk/fsl/fslwiki/GLM). Dual regression itself may take a day or more to complete depending on the number of group ICA components to be analyzed, number of subjects within each group, and complexity of the experimental design (e.g., number of contrasts). The final stage of dual regression analysis (stage 3) will generate t-statistic (tstat) maps for each component that correspond to each group contrast in chosen design matrix. Output files are named after corresponding component and contrast numbers (e.g., component X, contrast 1 would be named *dr_stage3_ic000X_tstat1*; dr, dual regression; ic, independent component). Additionally, a file reflecting the average of all brains (*mask.nii.gz*) can be found within the dual regression output directory, and will be used in subsequent steps. Ultimately, results of dual regression will be entirely study-dependent, and are arguably best illustrated in the setting of a study with the added context of experimental groups and hypothesis.

**Step 12. Prepare T-Statistic Maps for Gaussian Mixture Modeling**. Subsequent processing steps require output files from dual regression to be (a) in standard space (i.e., have same dimensions as anatomical template) and (b) masked (i.e., contain only brain voxels, and exclude all non-brain voxels). Only group ICA components identified as “networks of interest” (see Step 10: Evaluate Group-Level Components to Identify Networks of Interest) are evaluated further with Gaussian mixture modeling. Accordingly, only t-statistic maps corresponding to selected components need be prepared at this step. Use the *fslsplit* command used previously during preprocessing to remove artefactual components.

a. **Alter Dimensions of T-Statistic Maps**. Output files from dual regression (t-statistic maps and mask file) must also have the same dimensions as anatomical template in order to be correctly processed in subsequent steps. One can alter dimensions of mask file (mask.nii.gz) and selected t-statistic maps (e.g., dr_stage3_ic0001_tstat1.nii.gz) to match anatomical template by using the *flirt* command in the following format:

$$\begin{array}{l} flirt\;-\text{in}<\text{​dr}\_\text{stage3}\_\text{ic0001}\_\text{tstat1​}>\\ \;-\text{out}<\text{​ic01}\_\text{tstat1​}>-\text{ref}<\text{​anat}\_\text{brain}>\\ \;-\text{applyxfm} \end{array}$$

Option –in designates input t-stat map (dr_stage3_ic001_tstat1) and -out designates output t-stat map with new dimensions. Option -ref designates anatomical template file (anat_brain) as the “reference” image, from which the new dimensions of t-stat map are determined. Option –applyxfm for “apply transformation” refers to the projection of t-statistic maps into this new dimensional space corresponding to reference file. Importantly, this step does not involve registration of the t-statistic map to anatomical template (as image registration can only be performed between structural images, and not statistical maps). As a last step, dimensions of the mask file generated by dual regression (mask.nii.gz) should also be altered to match anatomical template.

**Step 13. Threshold T-Statistic Maps Using Gaussian Mixture Modeling**. Following dual regression, output t-statistic maps are thresholded to identify clusters (i.e., spatially contiguous regions of voxels) of significant brain activity. Cluster-based analysis can be statistically advantageous compared to alternative methods analyzing individual voxel units, improving SNR within each unit and reducing the number of hypotheses tested (Pendse et al., 2009). There are a number of approaches to cluster analysis, including the popular threshold free cluster extent (TFCE) (Smith and Nichols, 2009) that is not without its limitations (see report by Woo et al., 2014). Here, we will describe a generalized false discovery rate (FDR) approach. Specifically, Gaussian mixture modeling (GMM) is used to generate probability density histograms for each spatial map of z-scores by estimating the posterior probability that activity in a given voxel is significantly modulated by associated timecourse (Pendse et al., 2009). The alternative hypothesis is tested at P > 0.5 for “activation” (neuronal signal) vs. null (non-neuronal background noise). In other words, significant brain activity is defined when the probability of reflecting neuronal signal exceeds the probability of reflecting non-neuronal noise. The null hypothesis is estimated adaptively from the data as a mixture of Gaussians, from which voxel membership is estimated as one of three classes: “deactivation,” ”activation,” and “null” distributions. Generated histogram will allow for meaningful thresholding of t-statistic maps prior to cluster analysis (next step). For more detail on methodology, refer to Pendse et al. (2009). Thresholding via GMM can be achieved within Matlab.

**Figure 9:**
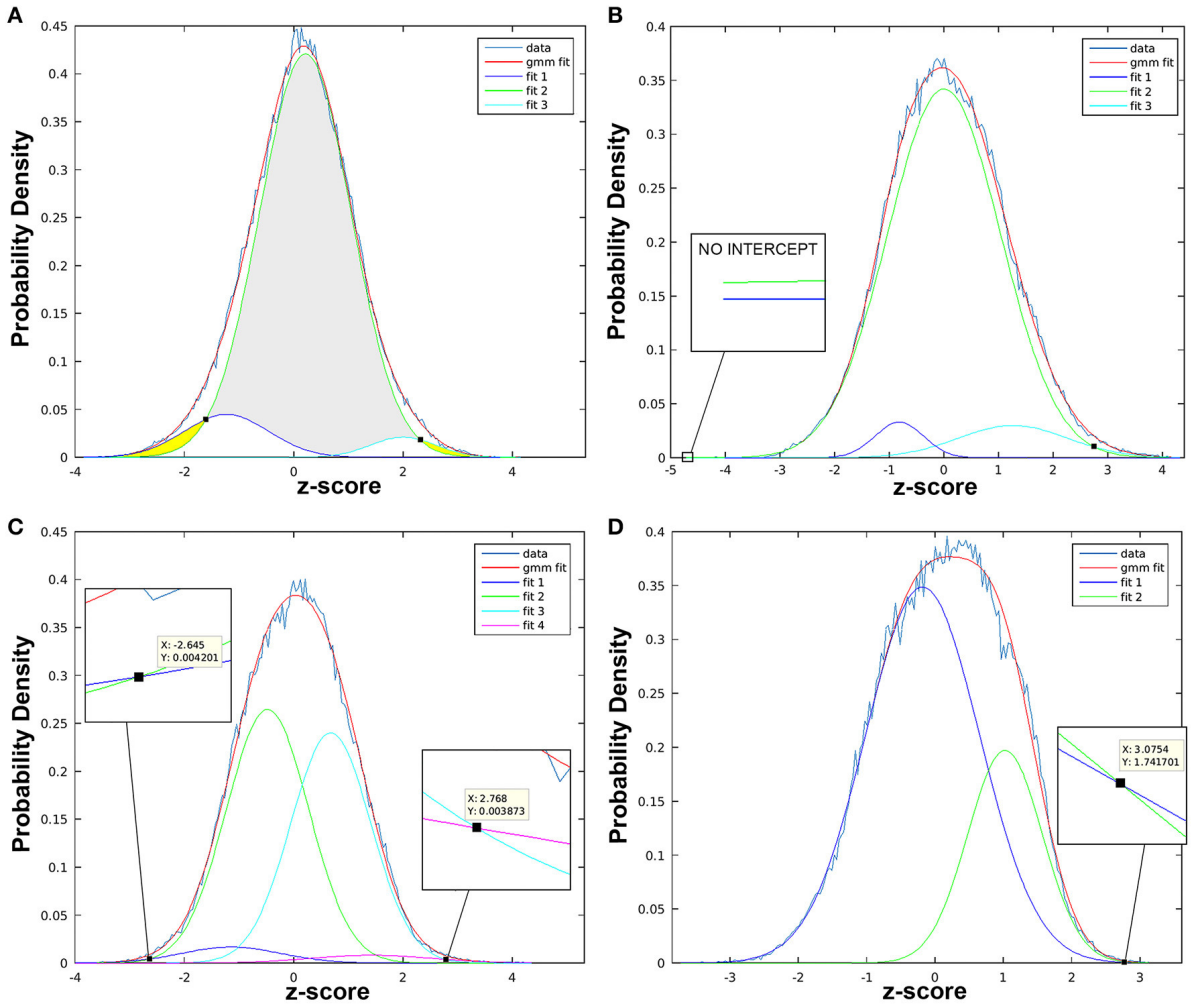
Statistical thresholds identified via Gaussian mixture modeling. Figure illustrates variable appearance of Gaussian mixture modeling (GMM) results using four t-statistic maps from different components **(A–D)**. Histogram of z-scores is shown for each t-statistic map (*data* line), modeled as the full mixture of Gaussians density (*gmm fit* line), as well as by distinct Gaussian sub-distributions according to class (i.e., “null,” “activation,” “deactivation”). Probability density (y-axis) is used to determine z-score threshold(s) of statistical significance (black squares). **(A)** Example of more straightforward results, in which three fit curves are taken to be “deactivation” (*fit 1*, left-shifted with negative z-scores; dark blue line), “null” (*fit 2*, centrally localized near zero; green line), and “activation” (*fit 3*, right-shifted with positive z-scores; turquoise color line). Gray region indicates z-values with statistically insignificant BOLD activity, while yellow regions highlight z-scores beyond identified thresholds exhibiting statistically significant BOLD activity. Intercepts between fit 1 (or fit 3) with “null” (fit 2) curve, is marked with small black square. **(B)** Example of t-statistic histogram with only one significant positive threshold (black square intercepts), despite presence of both positive (fit 3) and negative (fit 1) distributions. Probability density of the latter never surpasses the null (fit 2), confirmed with zoomed in view (no intercept). **(C)** Example of “split null” distribution, with the null class modeled by fit 2 and 3. Note, only the z-score furthest from zero is considered the threshold: negative threshold (z-score = −2.645) is described when fit 1 > fit 2 (not when fit 1 > fit 3), while positive threshold (z-score = 2.768) is described when fit 4 > fit 3 (not when fit 4 > fit 2). **(D)** Example for more challenging interpretation of results when fit 2 (green line) can be described as right-shifted (suggestive of “activation” class) with large volume (suggestive of “null” class).

- Identify Significance Thresholds: As a first step, one should create a new folder to run the analysis in (e.g., *GMM_Component#_tstat*). Next, use the following Matlab script with inserted name of t-statistic file to be processed:
  threshld_gmm('../tstat1_ic00.nii.gz','../mask.nii.gz',[],1000,1)
  When the script is finished running, a histogram (Figure 9) with several distributions will appear, where the x-axis shows z-scores and y-axis provides a measure of probability. Typically, null distributions are modeled by one or two (“split null”) Gaussian curves (larger volumes, centered near zero), while activation and deactivation curves are typically modeled as solitary Gaussian curves (with smaller volumes), skewed to the right and left of null distribution, respectively (Pendse et al., 2009). Non-Gaussian distributions skewed to the left or right of the null curve are described as “negative” and “positive,” respectively. This simply indicates opposite polarity of signal modulation, resulting from sign ambiguity (i.e., scaling indeterminacy) in the ICA model. Importantly, sign ambiguity precludes fixed interpretations of polarity (e.g., positive distributions could reflect increased or decreased brain activity). Thresholds are defined as z-scores at which non-null distributions (positive and/or negative) intersect with null curve. Record thresholds for each t-statistic image, as these will be used to perform cluster analysis in the subsequent step.
- Use Thresholds to Perform Cluster Analysis: Statistical thresholds identified in GMM histogram of each t-statistic map will now be used to identify regional patterns of brain activity via cluster analysis. Within the GMM folder (*GMM_Component#_tstat*), create an additional two folders named “Positive” and “Negative,” referring to the opposing polarities of non-Gaussian distributions. Separately, within each directory, use the following in-house Matlab script with appropriate threshold values to perform cluster analysis:
  postprocess_memx_ordered_maxima
  The statistical spatial map can be considered a function with peaks (maxima) and valleys (minima). The goal of the clustering procedure is to identify peaks by growing connected clusters around each maximum in the first step. In the next step, all voxels above the chosen threshold are assigned to clusters from Step 1 using a minimum cluster distance. This step may require anywhere from 5 minutes to several hours, depending on the number of clusters present.

**Step 14. Anatomical Allocation of Brain Clusters (Creation of Tables)**. The final product of this step is a summary report of cluster analysis, including each identified cluster's spatial location (corresponding brain coordinates), volume (number of composite voxels) and peak statistical values (measure of brain activity). This is achieved after active (or de-active) voxels are associated with a particular cluster. Each cluster is assigned to a brain region based on the coordinate of its maxima, as determined using the in-house Paxinos and Watson (1998) atlas-based rodent template (see Figures 7 and 8), and the volume of the cluster is calculated. One should review the tables of all clusters to identify physiologically relevant networks showing significant differences between experimental groups that will inform subsequent interpretations of functional connectivity. Such networks should be converted into text files and presented as tables. For examples of cluster tables, refer to published manuscripts (Becerra et al., 2011b; Bajic et al., 2016). During this process, it is also advisable to survey clusters overlaid on corresponding group ICA spatial maps (e.g., using FSLView).

## Timing

Making sure that all the software programs are operational is the first requisite for time-efficiency. As previously mentioned, analyses presented herein used a reconfigured Apple Mac Pro 6-Core Intel Xeon E5 (2.70 GHz) with 64 GB RAM to run OS Ubuntu 14.04.1. Individual preprocessing steps (Steps 1–3, 5), as well as data cleaning (Step 8) will require several minutes per individual subject file. As previously listed, time required to complete. Manual brain extraction (Step 4) of anatomical and fMRI images is the most time consuming step. It will depend on acquired image resolution (e.g., number of slices) as well as the skill of the operator. It may range from one to several hours. Step 6: Melodic Interface (during preprocessing, including single-session ICA analysis) will scale with the number of subjects included in the analysis. In other words, the more fMRI data files analyzed at one time, the longer the analysis. On average about 1 hour for an individual animal subject. This will also be true for group ICA analysis (Step 9: Network Detection via Group ICA), with increasing amounts of data to be processed costing higher time penalties. Similarly, remaining Steps 10–14 may take several days to complete.

## Troubleshooting

### Motion censoring (data scrubbing)

Essentially all fMRI studies are susceptible to some degree of motion artifact. It is now known that commonly used regression techniques (e.g., regression of realignment parameters) are inadequate to remove motion-induced artifact, and that motion residuals introduce systematic biasing effects on measures of functional connectivity in a distance-dependent manner (Power et al., 2012; Satterthwaite et al., 2012; Van Dijk et al., 2012; Kundu et al., 2013). Further, motion-related artifacts are known to arise from micromovements less than a few tenths of a millimeter (Power et al., 2012). Thus, while particularly important in cases of imaging awake animals (see previously published work: Figure 1 in (Becerra et al., 2011b); and Figure 2 in Becerra et al., 2011a), motion must be addressed when imaging anesthetized animals as well (see Figure 3 in Bajic et al., 2016). Typically as part of data quality assessment, input data files <hdrfile> should be visually inspected (e.g., using *fslview* command in Terminal) for obvious and pervasive motion artifacts throughout each BOLD time-series. As previously described in Step 6: Melodic Interface, MCFLIRT motion correction is used during preprocessing to correct for changes in head position throughout scan. Realignment parameters reported by MCFLIRT are useful for qualifying (e.g., sharp or gradual) and quantifying (e.g., isolated spike or pervasive) motion present in fMRI time-series. Graphical results depicting MCFLIRT realignment parameters are shown in each rodent's Melodic report (report.html). Graphs should be closely inspected for the extent of motion corruption. Currently, motion censoring (i.e., “data scrubbing”) is the most effective and conceptually simple method for motion-related artifact removal (Power et al., 2014), whereby volumes with motion exceeding defined motion parameter thresholds are discarded from the dataset. Unsalvageable animal time-series should be excluded from subsequent group ICA analysis (Step 9: Network Detection via Group ICA), as unrecognized motion can introduce false significance of identified networks and lead to erroneous interpretation of results. To perform motion censoring manually, use command:

fslsplit<​hdrfile​><​output basename​>−t

Option “-t” will split the 4D fMRI time-series (hdrfile) into separate 3D volumes with designated basename (e.g., volume_00##), after which motion-corrupted volumes can be deleted. To recombine remaining volumes into 4D time-series, use command:

fslmerge<​hdrfile_scrubbed​><​file1 file2…​>

Here, input files <file1 file2…> represent all volumes to be retained in new “scrubbed” time-series (hdrfile_scrubbed). Additional important notes on employing data scrubbing technique:

#### Attention to differences in volume numbering

When looking at the online Melodic report.html, MCFLIRT graphical result begin numbering volumes at “volume 1” (Step 7a: Pre-stats tab) and components begin numbering at “component 1” (see Step 8: Data Cleaning). This is in contrast to FSL software and actual Melodic output files, which begin volume numbering at “volume zero” and component numbering at “component zero.”

#### Limitations of the technique

(i) Minimum time-series length. Correlation estimates become more accurate and statistically robust with increasing numbers of volumes included in analysis, and increasingly noisy with decreasing numbers of volumes. Thus far, the field has typically considered 5 minutes of resting-state data per subject adequate to achieve network stabilization in humans (Van Dijk et al., 2010), however, there is currently no established limit on the amount of data that must be retained in a given fMRI file (Power et al., 2015). (ii) Controlling for time-series length. Depending on the analysis (e.g., multivariate pattern analysis), it is advisable to control for time-series length in order to maintain equal degrees of freedom across subjects (Power et al., 2014). Statistical bias may be otherwise introduced due to the direct relationship between extent of motion censoring and amount of motion present in scanned data. This is particularly relevant for rodent models of higher-moving animals (e.g., Parkinson's disease). Time-series length can be controlled at the individual and/or group level, such that all files and/or all experimental groups entering final analysis contribute equal proportions of BOLD signal. **Note** that at the individual level, all rodent files are reduced to minimum time-series length found in the sample. Alternatively at the group level, all experimental groups are made to contain the same amount of data, but allow individual time-series lengths to vary. In other words, for group-level analyses (e.g., average seed-maps) in samples containing *hundreds* of subjects, differences in the amount of data contributed by each subject's file appears to have little impact on final results (Power et al., 2014). In such cases, there may be no need to control for time-series length. (iii) Preserving temporal contiguity. Time-series should ideally retain temporal contiguity (i.e., disparate lengths of volumes should not be rejoined). Removal of successive volumes has been shown to remove more important information about BOLD signal than if the same number of non-contiguous volumes were removed (Power et al., 2015). Importantly, temporal contiguity is required for rs-fMRI data preprocessing using ICA-based artifact removal, which relies heavily on correlated temporal covariance across a period of minutes. Accordingly, only motion contamination at the beginning or end of time-series can be removed (while still controlling for degrees of freedom). If time-series temporal contiguity is retained during motion censoring, scrubbed data can undergo Melodic preprocessing run (Step 6: Melodic Interface and Step 7: Review Melodic Report) and subsequent data cleaning via ICA-based artifact removal. If temporal contiguity is forfeited during data scrubbing in order to salvage subject datasets, additional data cleaning via ICA-based artifact removal should not be performed. In this event, automated data scrubbing based on defined motion thresholds can be achieved using alternate software such as the Data Processing Assistant for Resting-State fMRI (DPARSF) V4.0 Rat module (Schwarz et al., 2006) based on Statistical Parametric Mapping (SPM8; www.fil.ion.ucl.ac.uk/spm) and toolbox for Data Processing & Analysis of Brain Imaging (DPABI Yan et al., 2016, http://rfmri.org/DPABI). Best strategy to minimize residual artifacts in the data will ultimately be informed by results of quality assessment.

### Registration

Being that animals slightly differ in brain size, individual input rs-fMRI data are registered to a standard space template during brain network analysis using group ICA to facilitate comparisons across all animals within a group (Step 9: Network Detection via Group ICA). Registration of all functional images to a common coordinate system is a pre-requisite for group ICA, and accurate alignment is critically important for valid final analysis. Inaccurate registration can compromise statistical analyses performed thereafter, resulting in erroneous final results from which no valid conclusions can be drawn. However, poor registration may be salvageable, and several avenues exist to fix suboptimal registration. One option would be to rerun FLIRT using the *example_func* file (output by Melodic) as the initial functional image for registration. Alternatively, normalization to a standard template may be improved with non-linear transformation using FNIRT (Jesper et al., 2007). In human subjects, non-linear registration risks warping fMRI data due to variability in cortical folding. However, gyral folding in the rodent brain is less substantial, reducing the risk of such warping. Lastly, poor registration may be the result of magnetic field inhomogeneity that presents as BOLD distortions (e.g., stretching or warping) in the fMRI image. Correction methods to rectify susceptibility-induced distortion are available, including use of top-down distortion correction (Holland et al., 2010), a self-field map (Jezzard and Balaban, 1995; Cusack et al., 2003; Zerbi et al., 2015), or a mean field map (Gholipour et al., 2008). The latter two options require forethought, as an additional scan must be acquired at the time of data acquisition.

## Anticipated results

**Figure 10:**
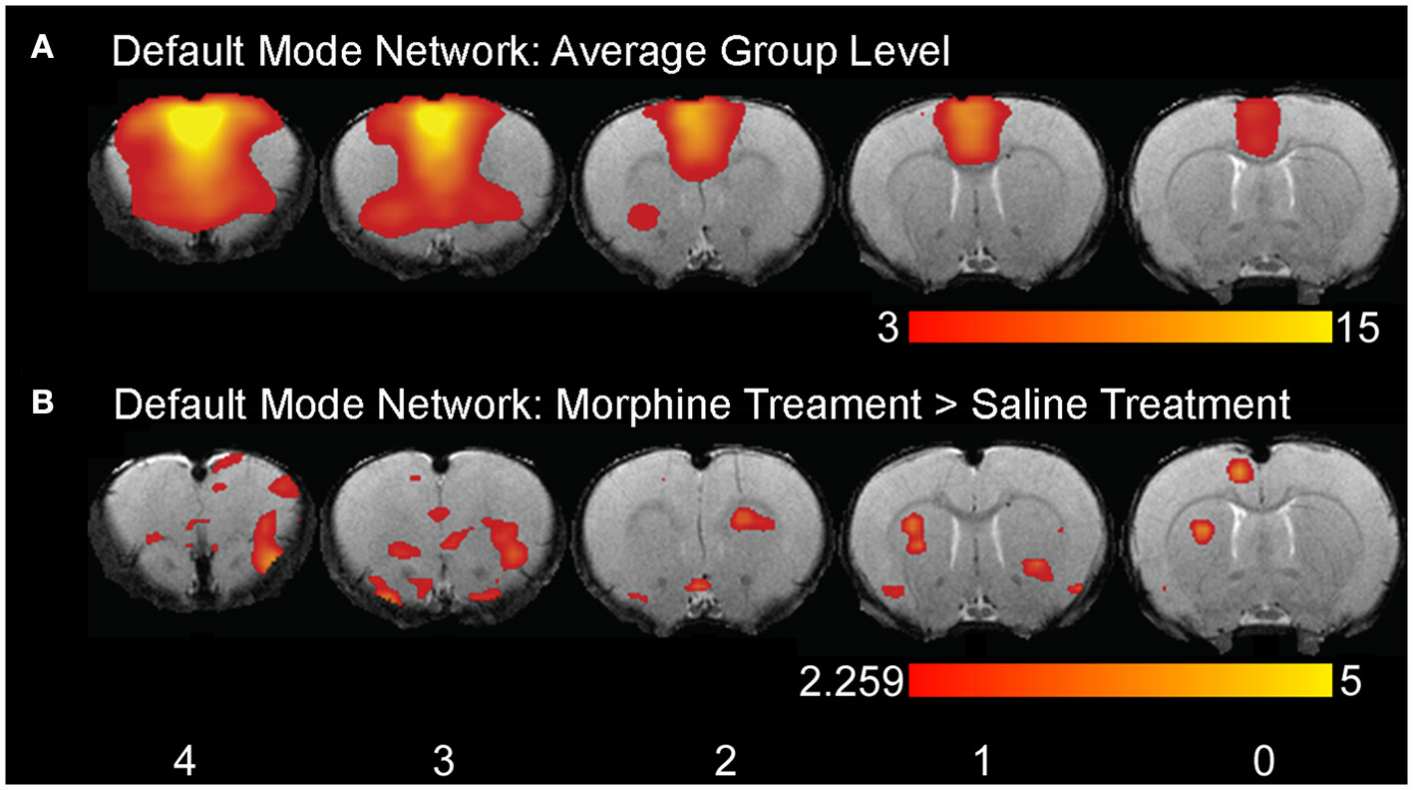
Example of rodent resting-state network and cluster analysis. **(A)** Example of a group ICA spatial map. Specifically, this row of images illustrates the *Default Mode Network* at the group level. **(B)** Example of group-level differences between adult rat ICA spatial maps. It is an example of group level differences between rats previously treated with morphine or saline in early neonatal period. Melodic report spatial maps are presented as z-scores superimposed on mean functional image in radiological convention (right side of image corresponds to left side of brain). The numbers at the bottom (below each coronal section) refer to distance from Bregma (in mm).

After running group ICA (Step 9), Melodic will output enforced dimensionality (e.g., 40 components) that can be visualized in Melodic report web browser. These components can be used to distinguish brain networks from artefactual components (see Figure 5 for examples). However, networks of interest should be thresholded prior to presentation. Spatial correlation between each group-level spatial map and template networks (Step 10: Evaluate Group-Level Components to Identify Networks of Interest) produces a set of Pearson's R values. Networks with an R value greater than 0.2 can be processed further (Figures 6A,B). Additionally, networks that exhibit neurobiologically plausible spatial distributions should also be included in remaining analyses (Figure 6C). Dual regression (Step 11) creates a number of t-statistic maps that correspond to between-group contrasts (e.g., contrast 1 = tstat1). In other words, for each experimental group included in analysis, dual regression will generate a set of associated t-statistic images that each correspond to a given group-level ICA component. Gaussian mixture modeling (Step 13a) identifies thresholds of statistical significance in t-statistic maps for each component, with thresholds typically ranging from 1 to 4. Identified thresholds are subsequently used to perform cluster analysis (Step 13b), which produces text files that display cluster location (brain coordinates), volume (number of constitutive voxels) and peak statistical significance (measure of connectivity strength). These are subsequently organized into tables that describe brain clusters showing significant connectivity (Step 14). All of the above files can be viewed using FSLview (https://fsl.fmrib.ox.ac.uk/fsl/fslwiki/FslView) or MRIcron (http://www.mccauslandcenter.sc.edu/mricro/mricron/). Refer to Figure 10 for an example illustration of a final rodent brain networks.

## Author contributions

All authors contributed extensively to the work presented in this paper. DBa outlined the method manuscript, performed, and analyzed data, as well as wrote the manuscript. MC performed experiments, analyzed data, and wrote the manuscript. CM analyzed data and wrote the manuscript. DBo wrote the manuscript. LB set up the computing devices, designed analysis methodology, and wrote the manuscript. All authors agree to be accountable for presented content of the work.

### Conflict of interest statement

The authors declare that the research was conducted in the absence of any commercial or financial relationships that could be construed as a potential conflict of interest.

## Footnotes

^1^Fusion classifier refers to the strategic “stacking” of multiple independent classifiers in order to increase overall accuracy of predictive model with weighted predictions. In this way, shortcomings associated with any one classifier are compensated through the strengths of others (Salimi-Khorshidi et al., 2014).

## Abbreviations

BOLD: blood oxygen level dependent
DVARS: derivative of RMS variance over voxels
DPABI: Data Processing & Analysis of Brain Imaging toolbox
DPARSF: Data Processing Assistant for Resting-State fMRI toolbox
FAST: FMRIB's Automated Segmentation Tool
FASTMAP: Fast Automatic Shimming Technique by Mapping Along Projections
FD: framewise displacement
FDR: fast discovery rate
FEAT: FMRI Expert Analysis Tool
FIX: FMRIB's ICA-based X-noiseifier
FLIRT: FMRIB's Linear Image Registration Tool
fMRI: functional magnetic resonance imaging
FSL: FMRIB's Software Library
GIFT: Group ICA Of fMRI Toolbox
GMM: Gaussian mixture modeling
GUI: graphical user interface
ICA: independent component analysis
MCFLIRT: Motion Correction using FMRIB's Linear Image Registration Tool
MELODIC: Multivariate Exploratory Linear Optimized Decomposition into Independent Components
MRI: magnetic resonance imaging
PD: postnatal day
N3: non-parametric non-uniform intensity normalization
pICA: probabilistic independent component analysis
BET: Brain Extraction Tool
RMS: root-mean-squared
ROI: region of interest
rs-fMRI: resting-state functional magnetic resonance imaging
RSN: resting-state network
SCA: seed-based correlation analysis
SNR: signal-to-noise ratio
TE: echo time
TFCE: threshold free cluster extent
TR: repetition time
tstat: t-statistic.
